# Solvatochromism and Click Reactivity of Yellow Tetrazines

**DOI:** 10.1021/acs.bioconjchem.6c00097

**Published:** 2026-08-27

**Authors:** Patrick Keppel, Jakob Raffler, Hannes Mikula, Dennis Svatunek

**Affiliations:** Institute of Applied Synthetic Chemistry. TU Wien Getreidemarkt 9, 1060 Vienna, Austria

## Abstract

Hydroxypyridyl-substituted 1,2,4,5-tetrazines have recently been developed as advanced scissors for bioorthogonal click-to-release chemistry. In contrast to the otherwise characteristic pink color of 1,2,4,5-tetrazines, these compounds were observed to give yellow solutions in methanol and buffered aqueous media. This study shows that the solvatochromism of hydroxypyridyl-tetrazines has its origin in varying protonation states of the phenolic OH group and reveals the impact of deprotonation on bioorthogonal click reactivity. We demonstrate that the rate of the Diels–Alder cycloaddition is significantly affected by the pH of the aqueous solution, thereby providing critical insights for the development of molecular tools for bioorthogonal bond cleavage.

## Graphical abstract

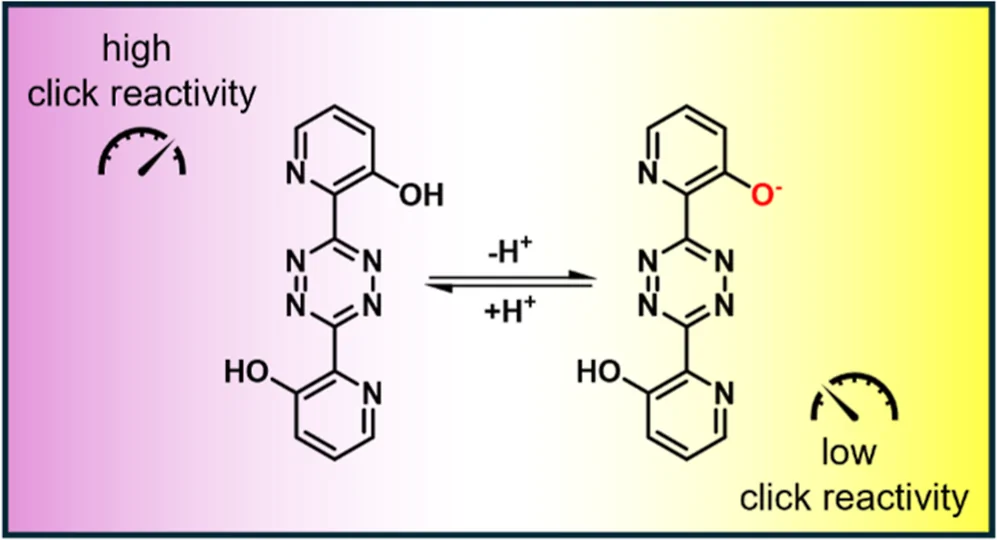

## Introduction

Since its discovery in 2008,^1^ the inverse-electron-demand Diels–Alder (IEDDA) reaction between 1,2,4,5-tetrazines (Tz) and strained dienophiles has become one of the most powerful bioorthogonal chemistries. Common dienophiles include *trans*-cyclooctenes (TCO), bicyclo[6.1.0]­nonyne (BCN), norbornenes, and cyclopropenes, which display a broad range of reactivities.^2^ Reported second-order rate constants span several orders of magnitude, ranging from approximately 1 M^–1^s^–1^ for norbornene derivatives^3^ to 10^2^–10^3^ M^–1^s^–1^ for strained cyclooctynes such as BCN^4^ and up to 10^4^–10^6^ M^–1^s^–1^ for highly reactive TCOs.^5–7^ Among these, TCOs are the most widely used owing to their exceptionally high reaction rates. In 2013, Robillard and co-workers demonstrated that this reaction can be applied not only for fast ligation but also to achieve selective cleavage of TCO conjugates triggered by the initial cycloaddition (click-to-release).^8^ *trans*-Cyclooctenes functionalized with a carbamate group in the allylic position are used, which upon reaction with a Tz yield a dihydropyridazine intermediate that undergoes tautomerization, followed by 1,4-elimination leading to the release of the carbamate group (Figure 1A).^8^ The discovery of Tz/TCO bond-cleavage chemistry enabled the development of bioorthogonal approaches for targeted drug delivery and the click-triggered activation of TCO-caged prodrugs, thereby advancing bioorthogonal chemistry into clinical application.^9–11^ In 2018, Carlson et al. demonstrated that controlling and accelerating the tautomerization step is crucial for ensuring fast and complete cleavage.^12^

**Figure 1:**
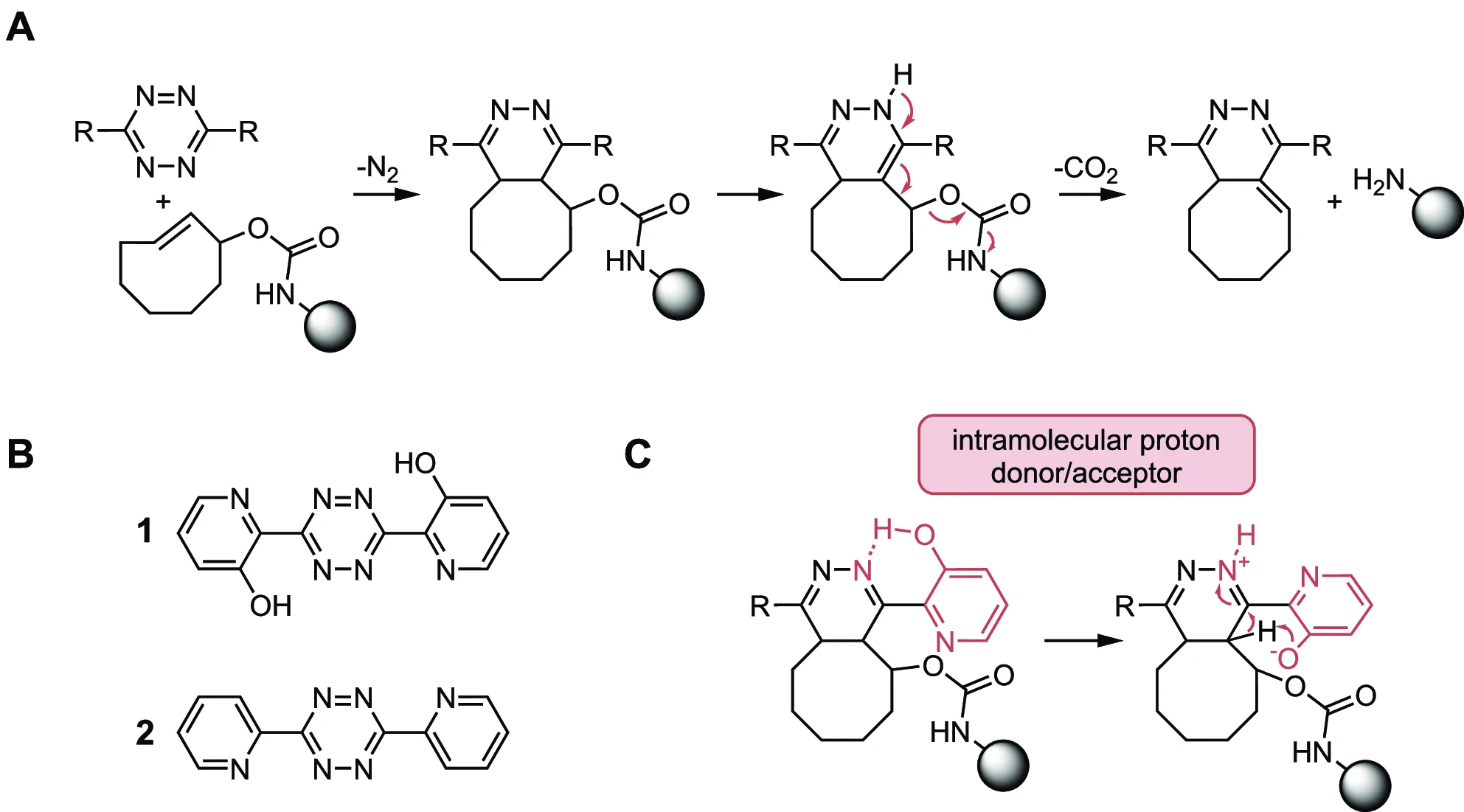
(A) Mechanism of the click-to-release reaction between tetrazines and *trans*-cyclooctene modified at the allylic position with a caged payload that is released via tautomerization and 1,4-elimination; the resulting dihydropyridazine shown can subsequently rearomatize or tautomerize (not shown). (B) Bis­(hydroxypyridyl)-Tz **1** and its nonhydroxylated analog **2**. (C) 3-Hydroxy-2-pyridyl substituents act as intramolecular proton donors and acceptors, facilitating tautomerization and subsequent elimination.

We recently developed hydroxylated pyridyl-Tz, more specifically 3-hydroxypyridin-2-yl-substituted tetrazines, such as the symmetrical tetrazine **1** (Figure 1B) as bioorthogonal scissors for accelerated click-to-release.^13^ In general, highly reactive pyridyl-substituted tetrazines (e.g., Tz **2**), frequently used for fast click ligation, achieve only limited release.^8,14–16^ By introducing phenolic OH functionalities as intramolecular proton-exchanging groups (Figure 1C), we transformed these scaffolds to facilitate high click reactivity and efficient release. Importantly, the same principle has been extended to other systems as well: sulfonated phenol-tetrazines have also been shown to promote efficient cleavage, highlighting that proton relay-assisted elimination is a generalizable strategy for designing effective click-to-release reagents.^17^

Unexpectedly, while hydroxylated pyridyl-tetrazines appear pink to red in solid form and when dissolved in aprotic organic solvents (e.g., acetonitrile), aqueous and methanolic solutions exhibit a yellow to orange color (depending on the concentration).^13^ Here, we explore the origin of this solvatochromism and its impact on click reactivity.

## Results and Discussion

Comparing the structure of “yellow” tetrazine **1** with the nonhydroxylated derivative **2** (pink in solid form and in solution) indicates that the OH group significantly influences the spectroscopic properties.

Tetrazines typically absorb visible light at a wavelength of around 530 nm, which is the reason for their pink appearance. This absorbance is relatively weak compared to the stronger absorbance observed in the ultraviolet region.^18^ The absorption at 530 nm is highly consistent across various 1,2,4,5-tetrazines with different substituents^19^ and frequently utilized to study kinetics of click reactions by monitoring its disappearance over time.^18,20–24^

In contrast to the bis-pyridyl-substituted tetrazine **2**, which maintains its characteristic pink color in solvents such as acetonitrile, methanol, and phosphate-buffered saline (PBS, pH 7.4), the 3-hydroxy-2-pyridyl-substituted tetrazine **1** exhibits a pink color when dissolved in acetonitrile but shifts to yellow in methanol and PBS (Figure 2B). Spectra in additional solvents can be found in Supporting Information Figure S1. Notably, the yellow color is significantly more intense than the pink, with the methanol solution displaying a more pronounced color than the aqueous PBS solution (see Supporting Information Figure S2).

**Figure 2:**
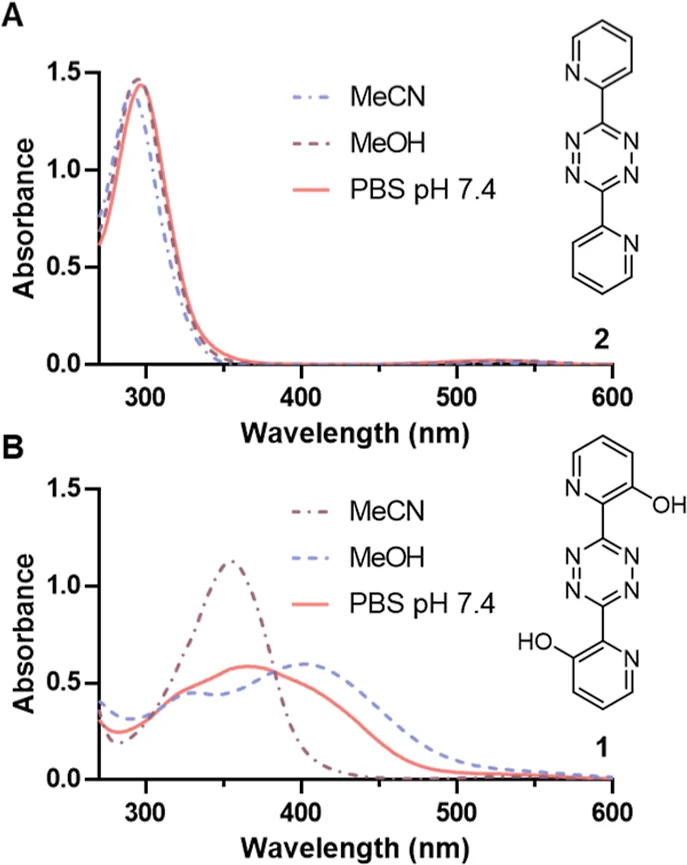
UV–vis spectra of (A) bis-pyridyl-Tz **2** and (B) bis­(hydroxypyridyl)-Tz **1** (50 μM) in acetonitrile, methanol, and PBS (pH 7.4).

The UV–vis spectra of compounds **1** and **2** (Figure 2) reveal that the characteristic absorbance at 530 nm is present in all instances. However, for compound **1** in methanol and PBS, there is a noticeable bathochromic shift of the strong absorbance typically observed in the UV region. This shift is responsible for the yellow appearance of compound **1** when dissolved in these solvents.

Based on the UV–vis analysis of tetrazines **1** and **2** in different solvents, we proceeded to investigate the pH dependence of the observed UV–vis absorbance. Therefore, we used citrate-buffered aqueous solutions at pH values ranging from 4.0 to 8.5. For pyridyl-tetrazine **2,** we observed no significant change in absorbance at different pH values, as detailed in the Supporting Information (see Figure S4). In contrast, bis­(hydroxypyridyl)-tetrazine **1** exhibited a clear pH dependence, including the presence of an isosbestic point (Figure 3). At low pH the absorbance shifted to the UV region, while higher pH values led to absorbance extending into the visible spectrum, specifically around 400 to 500 nm. Additionally, the color change was found to be reversible (Supporting Information Figure S5).

**Figure 3:**
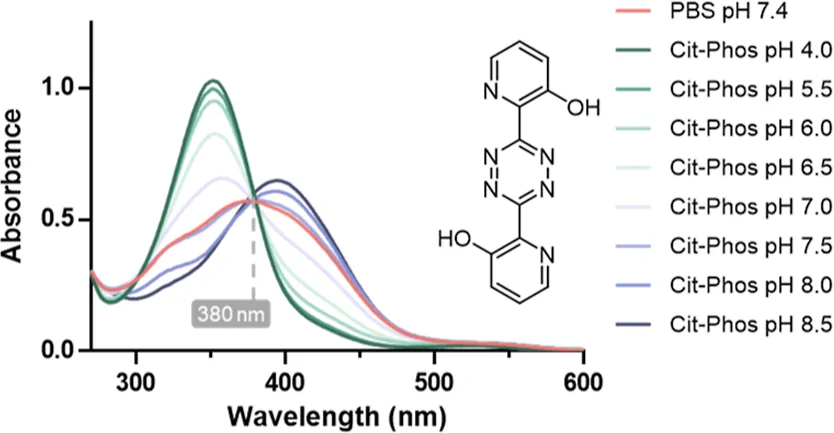
pH dependence of the UV–vis absorption of Tz **1** (50 μM in citrate-phosphate buffered solution), with an isosbestic point at 380 nm.

Based on these data, we hypothesized that one of the hydroxyl groups in compound **1** is partially deprotonated at pH values around 6 and above. This deprotonation causes a bathochromic shift, leading to the observed yellow color. To support this hypothesis, UV–vis spectra were computationally modeled using time-dependent density functional theory (TD-DFT). Calculations were performed in ORCA version 5.0.4,^25^ using the M06-2X functional and the def2-TZVP basis set, with the conductor-like polarizable continuum model (CPCM) for water as treatment of the solvent. A detailed description of the computational methods can be found in the respective section at the end of this manuscript.

Figure 4 shows the calculated spectra in CPCM water of Tz **1** and its deprotonated form **[1–H]** ^**–**^. In agreement with experimental data, deprotonation causes a clear bathochromic shift from 317 to 400 nm.

**Figure 4:**
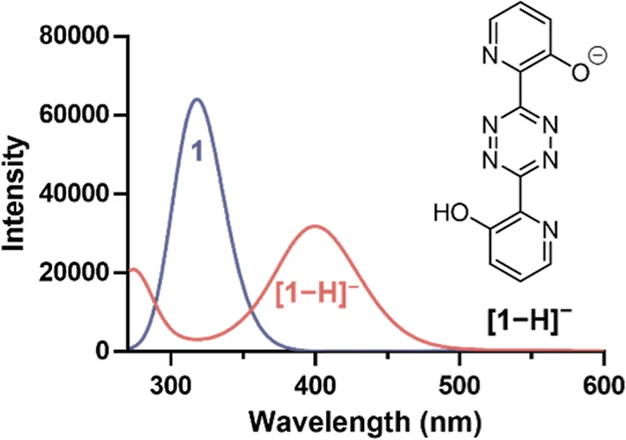
TD-DFT calculated UV–vis spectra of **1** and **[1–H]** ^**–**^.

Hence, the pH-dependent appearance and the solvatochromism of bis­(hydroxypyridyl)-Tz **1** can be attributed to the deprotonation of the phenolic OH functionality. While our TD-DFT analysis is consistent with monodeprotonation as the dominant species under the conditions studied, double deprotonation at strongly alkaline pH cannot be fully excluded. Our observations are further supported by previously reported p*K*_a_ measurements for **1** (p*K*_a_ 6.9 and 8.3).^17^ To test whether this effect arises from a single hydroxypyridyl group rather than a cooperative effect of both groups, we repeated the measurements using a derivative of **1** in which one of the hydroxypyridyl groups is replaced by a methyl group. This compound **4** exhibited comparable solvatochromism as **1** (Supporting Information Figure S6). In acidic aqueous media and in aprotic solvents, the hydroxyl group of these hydroxypyridyl tetrazines remains protonated, resulting in a pink color due to the characteristic absorption at 530 nm. At higher pH values and when dissolved in alcohols like methanol, the hydroxyl group is partially deprotonated, leading to a bathochromic shift of the strong UV absorption into the visible spectrum, resulting in yellow-colored solutions. Due to the significantly higher extinction coefficient of the 400 nm absorption band relative to that at 530 nm, the pink color is no longer observable; although the 530 nm band persists, it is masked by the much stronger absorption at 400 nm (ε up to 20-fold higher; see Supporting Information Table S2 and Figure 3).

We further hypothesized that the protonation state affects not only the optical properties but also the click reactivity of hydroxypyridyl-substituted tetrazines. Therefore, we conducted a computational investigation to compare the reactivity of Tz **1** and its deprotonated form **[1–H]** ^**–**^ in reactions with *trans*-cyclooctene. Calculations were carried out at the M06–2X/def2-TZVP level of theory with CPCM water as the solvent model. The free energy barrier for the reaction of Tz **1** with TCO was calculated to be 17.0 kcal/mol (Figure 5A). Notably, at the transition state, the hydroxyl group forms an intramolecular hydrogen bond with a nitrogen atom of the tetrazine. This interaction causes the nitrogen of the 3-hydroxy-2-pyridyl substituent to increase its distance to the adjacent tetrazine nitrogen (Figure 5B). Upon deprotonation of the OH group, the molecular structure adopts a different conformer due to repulsion between the negatively charged oxygen and the tetrazine nitrogen. In this transition state for the reaction between **[1–H]** ^**–**^ and TCO, the phenolate is thus positioned away from the nitrogen atom of the tetrazine, which forces the pyridyl nitrogen to be aligned instead.

**Figure 5:**
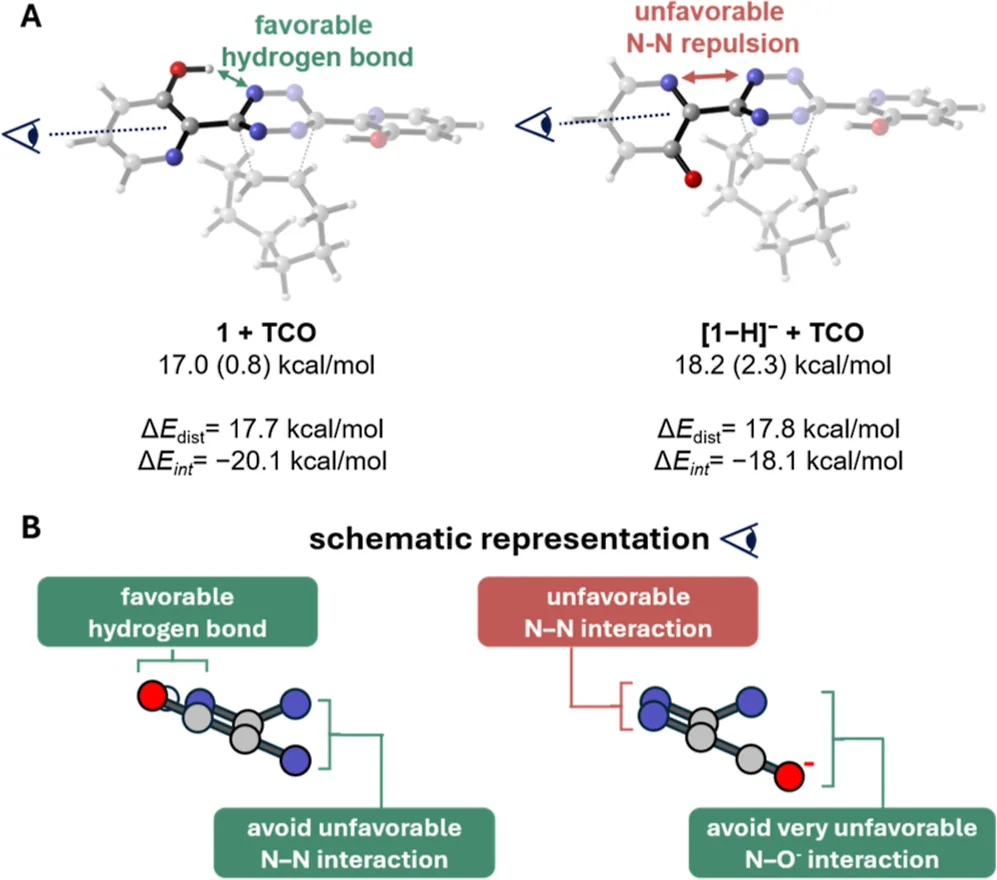
(A) Transition state geometries of **1** and **[1–H]** ^**–**^ with TCO. Free energy barriers and enthalpies of activation (in parentheses) are given in kcal/mol. Barriers were calculated using M06-2X/def2-TZVP CPCM­(water). Distortion/interaction energies were calculated using M06-2X/def2-TZVP. (B) Schematic representation of favorable and unfavorable interactions in the transition states of **1 + TCO** and **[1–H]** ^**–**^ + **TCO**.

To gain a deeper understanding of the factors leading to the higher barrier for **[1–H]** ^**–**^, we performed a distortion/interaction analysis (DIA). In this approach, the activation energy is decomposed into the energy required to distort the reactants from their equilibrium geometries (distortion energy, Δ*E*_dist_) to the transition-state geometry and the interaction energy between the distorted fragments (interaction energy, Δ*E*_int_).^26^ DIA has previously been used extensively to elucidate the origins of reactivity in tetrazine ligations.^24,27–29^

In the present case, the distortion energies are nearly identical for the protonated and deprotonated species, amounting to 17.7 and 17.8 kcal mol^–1^ for **1** and **[1–H]** ^**–**^, respectively. In contrast, the interaction energy is significantly less favorable for **[1–H]** ^**–**^ (−18.1 kcal mol^–1^) than for **1** (−20.1 kcal mol^–1^), accounting for the observed decrease in reactivity (Figure 5A). This weaker interaction can be traced to less favorable frontier molecular orbital interactions. Specifically, deprotonation raises the energy of the tetrazine LUMO+1, the orbital possessing the appropriate symmetry for the cycloaddition, from −2.0 eV in **1** to −1.5 eV in **[1–H]** ^**–**^. The resulting larger HOMO­(TCO)–LUMO+1­(tetrazine) gap reduces frontier molecular orbital interaction and leads to a less favorable interaction energy at the transition state.

To confirm these findings, kinetic measurements were performed to study the reactions of tetrazines **1** and **2** with the PEGylated TCO-derivative **3** (axTCO-PEG_4_, **3**).^30^

In acetonitrile, Tz **1** and **2** exhibited very similar reactivity, with second-order rate constants of 910 M^–1^s^–1^ and 790 M^–1^s^–1^, respectively (Figure 6A). In methanol, we observed a > 2-fold increase in reactivity for Tz **2**, while the rate constant for bis­(hydroxypyridyl)-Tz **1** decreased substantially to less than 20% of its value in acetonitrile. This observation, marked by the pronounced yellow color of the methanolic solution of Tz **1** indicating significant deprotonation, aligns with and clearly supports our computational findings.

**Figure 6:**
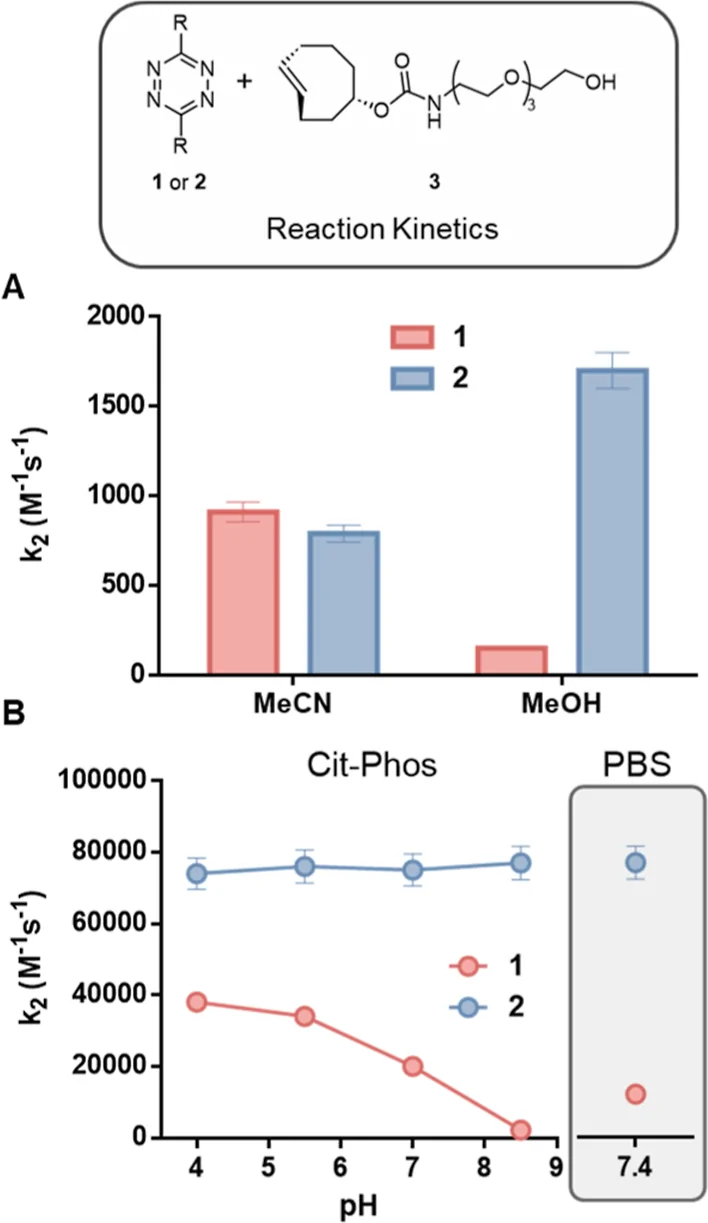
(A) Second-order rate constants for the reaction of Tz **1** and **2** with axTCO-PEG_4_ (**3**) in MeCN and MeOH at 37 °C. (B) pH dependence of the click reactivity of Tz **1** and **2** at 37 °C (Cit-Phos: citrate-phosphate-buffered solutions).

In PBS at pH 7.4, Tz **2** was found to be approximately 6 times more reactive than Tz **1** (Figure 6B). Further investigations using citrate-phosphate-buffered solutions across a pH range from 4.0 to 8.5 revealed no significant pH dependence of the click reactivity of pyridyl-tetrazine **2**. In contrast, we observed a marked pH dependence for Tz **1**, with second-order rate constants varying from nearly 38,000 M^–1^s^–1^ at pH 4.0 to 2,200 M^–1^s^–1^ at pH 8.5, which reflects a 17-fold difference in reactivity. Compound **4**, which bears only a single hydroxypyridyl group, showed a similar pH-dependent reactivity trend as Tz **1**, demonstrating that a single hydroxypyridyl group is sufficient to produce this behavior (Supporting Information, Table S3).

To rule out a contribution from oxygen-based redox chemistry, we repeated the kinetic measurements for the reaction between **1** and **3** in oxygen-free and oxygen-saturated solutions; no difference in reactivity was observed, further supporting a deprotonation-based mechanism (Supporting Information, Figure S9).

The pronounced pH dependence observed for **1** in Figure 6B directly mirrors the deprotonation equilibrium of its phenolic OH groups: the reactivity is highest in acidic media, where the OH groups remain protonated, and decreases monotonically as the pH is raised and the phenolate forms, in agreement with the higher computed barrier for**[1–H]^−^** and with pH-dependent UV–vis measurements. Importantly, this behavior is not a general feature of tetrazine ligations, since the rate constant of the nonhydroxylated control **2** remains unchanged across the entire pH range studied, in agreement with the literature.^22,31^ The marked, 17-fold reduction in click reactivity observed for **1** is therefore specific to the ionizable hydroxypyridyl substituent and does not reflect an intrinsic pH dependence of the tetrazine–TCO cycloaddition.

As *trans*-cyclooctenes are not the only dienophiles capable of reacting rapidly with tetrazines, we also measured the reactivity of **1** and **2** with *endo*-BCN-PEG_4_ (**5**). Here, Tz **2** showed a consistent rate constant of 1,400–1,500 M^–1^s^–1^ across the range from pH 4 to 8.5, whereas the reactivity of **1** decreased from 1,100 M^–1^s^–1^ at pH 4 to 110 M^–1^s^–1^ at pH 8.5 (Supporting Information, Table S3), demonstrating that this effect is also present for the commonly used cyclooctyne-based system BCN.

## Conclusion

Hydroxypyridyl-substituted 1,2,4,5-tetrazines have recently been identified as advanced bioorthogonal scissors for click-to-release reactions. This study elucidates the origin of the unexpected yellow color of aqueous solutions of these tetrazines. The observed solvatochromism is attributed to the deprotonation of the hydroxyl group, especially at higher pH and in basic protic solvents, leading to a bathochromic shift of the strong UV absorption into the visible spectrum.

In addition, we explored the impact of the protonation state on the click reactivity of hydroxypyridyl-Tz in bioorthogonal Diels–Alder reactions with *trans*-cyclooctene and BCN, revealing a connection between optical properties and reactivity. We found that deprotonation of the phenolic OH group results in a substantial decrease in reactivity due to a reduced frontier molecular orbital interaction. The yellow color can serve as an indicator of the protonation state in solution and, consequently, the click reactivity of hydroxypyridyl-Tz.

These findings underscore the importance of understanding the underlying mechanisms and the possible pH dependence of bioorthogonal reactions. Unraveling the impact of deprotonation on the click reactivity of hydroxyaryl-Tz paves the way for the design of advanced tetrazines for click-to-release chemistry. Specifically, Tz derivatives with an increased p*K*_a_ of the phenolic OH group are hypothesized to maintain high reactivity in aqueous solution and at more alkaline pH levels, broadening the scope of these compounds in bioorthogonal chemistry. Furthermore, the pronounced pH sensitivity of the reactivity of 3-hydroxy-2-pyridyl-substituted 1,2,4,5-tetrazines has the potential to enable the development of pH-responsive click reactions.

## Experimental Section

### Safety Statement

No unexpected, new, or unusually high safety hazards or risks were encountered during the experimental work described in this manuscript.

Unless otherwise noted, reactions were carried out under an atmosphere of argon in air-dried glassware with magnetic stirring. Air- and/or moisture-sensitive liquids were transferred via syringe. All reagents were purchased from commercial suppliers and used without further purification. 3,6-Bis­(2-pyridyl)­tetrazine (**2**) and *endo*-BCN-NHS carbonate were obtained from Sigma-Aldrich and BLDpharm, respectively. Tz **1** was synthesized as previously reported.^13^ Dry acetonitrile (MeCN) and methanol (MeOH) were obtained from Thermo Scientific, and dry DMF and DMSO from ACROS Organics. Dry ethanol was purchased from VWR. All dry solvents were stored under argon. Dichloromethane was dried using PURESOLV columns (Inert Corporation). For reversed-phase purification and HPLC analysis, HPLC-grade water was obtained by using a Purelab Chorus 1 water purification system (ELGA), and HPLC-grade MeCN was purchased from VWR. Solvents used for flash column chromatography were purchased from Donau Chemie AG (Austria). Column chromatography was performed using a Pure Excellence C-950 Chromatography System (BÜCHI). Reversed-phase column chromatography was performed using a FlashPure EcoFlex cartridge (C18, 40 g, BÜCHI) and preparative reversed-phase column chromatography was performed using a Kinetex AXIA LC column (C18, 5 μm, 100 Å, 100 × 30.0 mm, Phenomenex). ^1^H and ^13^C NMR spectra were recorded on a Bruker Avance UltraShield 400 MHz or Bruker Ascend 600 MHz spectrometer at 20 °C. Chemical shifts are reported in ppm (δ) relative to tetramethylsilane and calibrated using residual solvent peaks. Data are shown as follows: chemical shift, multiplicity (s = singlet, d = doublet, t = triplet, q = quartet, quint = quintet, m = multiplet, br = broad signal), coupling constants (*J*, Hz) and integration. Reaction progress was monitored by thin layer chromatography (Merck, silica gel 60, fluorescence indicator F254) or by HPLC-MS analysis.

HPLC analysis was performed on a Nexera X2 UHPLC system (Shimadzu) comprised of LC-30AD pumps, SIL-30AC autosampler, CTO-20AC column oven and DGU-20A5/3 degasser module. Detection was done using an SPD-M20A photodiode array detector, an RF-20Axs fluorescence detector, an ELS-2041 evaporative light scattering detector (JASCO), and an LCMS-2020 mass spectrometer (ESI/APCI). All separations were performed using a Waters XSelect CSH C18 2.5 μm (3.0 × 50 mm) column XP at 40 °C and a flow rate of 1.7 mL/min with 0.1% aqueous formic acid or ammonium formate buffer (2.5 mM, pH 8.4) and acetonitrile (gradient elution). Acidic HPLC conditions (acetonitrile/0.1% formic acid) 0 min: 5%, 0.15 min: 5%, 2.20 min: 98%, 2.50 min: 98%; Buffered HPLC conditions (acetonitrile/2.5 mM ammonium formate buffer, pH 8.4) 0 min: 5%, 0.15 min: 5%, 2.20 min: 98%, 2.50 min: 98%.

Citrate-phosphate buffers^32^ (10 mM) were prepared by diluting from stock solutions of 0.1 M citric acid and 0.1 M Na_2_HPO_4_. pH values were verified and adjusted as needed to within ±0.05 pH units by digital pH metering (Mettler Toledo SevenCompact pH meter S220, reference buffers: Mettler Toledo technical buffer solutions at pH 4.01, 7.00, and 9.21).

### Synthesis

#### Synthesis of axTCO-PEG_4_ (**3**)

axTCO-*para*-nitrophenol (axTCO-PNP) was synthesized according to a previously reported procedure.^33^ axTCO-PNP (210.8 mg, 0.72 mmol) was dissolved in dry DMF (300 μL). 2-(2-(2-(2-Aminoethoxy)­ethoxy)­ethoxy)­ethanol (209.8 mg, 1.09 mmol), dissolved in 600 μL dry DMF, and DIPEA (189 mg, 255 μL, 1.46 mmol) were added subsequently. The reaction mixture was stirred for 2.5 h at room temperature. The reaction was quenched with formic acid (55 μL, 1.46 mmol) in 1 mL H_2_O at 0 °C. The solvent was evaporated under reduced pressure and the crude product was purified by column chromatography (EcoFlex C18, H_2_O/MeCN gradient elution, 0.1% formic acid) yielding **3** (165 mg, 66%) as a colorless oil. ^1^H NMR (400 MHz, DMSO-*d*_6_): δ 7.12 (t, *J* = 5.8 Hz, 1H), 5.71–5.43 (m, 2H), 4.72 (dd, *J* = 10.5, 5.3 Hz, 1H), 4.56 (t, *J* = 5.5 Hz, 1H), 3.60–3.36 (m, 14H), 3.12 (q, *J* = 6.0 Hz, 2H), 2.35–1.96 (m, 5H), 1.88–1.39 (m, 4H), 1.18 (q, *J* = 13.0 Hz, 1H). ^13^C NMR (101 MHz, DMSO-*d*_6_): δ 155.8, 135.0, 131.5, 72.3, 69.8 (2C), 69.7, 69.6, 69.2, 68.4, 60.2, 40.5, 33.9, 32.2, 29.6, 27.4, 24.5. ESI-MS [M + H]^+^ *m*/*z* calcd. 346.22 for C_17_H_32_NO_6_^+^; found 346.15.

#### Synthesis of Methyl-hydroxypyridyl-Tz *(* **4** *)*

A mixture of 3-hydroxypicolinonitrile (295 mg, 2.5 mmol, 1 equiv) and zinc triflate (137 mg, 0.4 mmol, 15 mol %) was purged with argon and subsequently charged with MeCN (370 μL, 7.0 mmol, 2.9 equiv) and hydrazine monohydrate (720 μL, 14.7 mmol, 6 equiv). After stirring at 70 °C for 21 h, the reaction mixture was allowed to cool to room temperature, filtered through a glass frit, and washed twice with dry ethanol (2 mL). Ethanol (14 mL) and glacial acetic acid (25 mL) were added to the filtrate under cooling with a water/ice bath at 0 °C. Subsequently, an aqueous solution of NaNO_2_ (1.2 g, 16.8 mmol, 7 equiv in 12 mL water) was added dropwise over 5 min, resulting in a color change from yellow to red. After stirring for 20 min, the solution was extracted with CH_2_Cl_2_ and the combined organic phases were dried over Na_2_SO_4_, filtered, and concentrated under reduced pressure. The resulting red residue was purified by preparative reversed-phase column chromatography (Kinetex AXIA C18 LC column; H_2_O/MeCN gradient elution, 0.1% formic acid). Product-containing fractions were combined and extracted with CH_2_Cl_2_, and the combined organic phases were dried over Na_2_SO_4_, filtered, and concentrated to give **4** as red crystals (11 mg, 2%). ^1^H NMR (600 MHz, CDCl_3_): δ 11.07 (s, 1H), 8.54 (dd, *J* = 4.2, 1.5 Hz, 1H), 7.52 (dd, *J* = 8.4, 1.5 Hz, 1H), 7.48 (dd, *J* = 8.5, 4.2 Hz, 1H), 3.18 (s, 3H). ^13^C NMR (151 MHz, CDCl_3_): δ 167.8, 164.2, 157.6, 142.7, 131.7, 128.7, 127.4, 21.6. ESI-MS [M + H]^+^ *m*/*z* calcd. 190.07 for C_8_H_8_N_5_O^+^; found 190.04.

#### Synthesis of *Endo*-BCN-PEG_4_ (**5**)

A solution of *endo*-BCN-NHS carbonate (39 mg, 131 μmol, 1 equiv) in dry CH_2_Cl_2_ (1.3 mL) was cooled to 0 °C with a water/ice bath. DIPEA (69 μL, 393 μmol, 3 equiv) was added, followed by PEG_4_-NH_2_ (38 mg, 197 μmol, 1.5 equiv) as a solution in dry CH_2_Cl_2_ (200 μL) under vigorous stirring. After 30 min, the reaction mixture was diluted with CH_2_Cl_2_ (5 mL) and saturated aqueous NH_4_Cl solution was added. The mixture was extracted with CH_2_Cl_2_, and the combined organic phases were dried over Na_2_SO_4_, filtered, and concentrated under reduced pressure. The resulting crude residue was purified by preparative reversed-phase column chromatography (Kinetex AXIA C18 LC column; H_2_O/MeCN gradient elution, 0.1% formic acid) to give **5** as a pale yellow oil (43 mg, 90%). ^1^H NMR (600 MHz, CD_2_Cl_2_): δ 5.87 (br s, 1H), 4.11 (d, *J* = 8.1 Hz, 2H), 3.71–3.64 (m, 4H), 3.64–3.55 (m, 8H), 3.51 (t, *J* = 5.0 Hz, 2H), 3.31 (q, *J* = 5.3 Hz, 2H), 3.00 (s, 1H), 2.30–2.15 (m, 5H), 1.64–1.51 (m, 2H), 1.34 (p, *J* = 8.6 Hz, 1H), 0.98–0.86 (m, 2H). ^13^C NMR (151 MHz, CD_2_Cl_2_): δ 157.3, 99.2, 73.0, 71.1, 70.9, 70.8, 70.6, 62.9, 62.0, 41.4, 29.6, 21.8, 20.6, 18.4. ESI-MS [M + H]^+^ *m*/*z* calcd. 370.22 for C_19_H_32_NO_6_^+^; found 370.17.

#### UV–vis Measurements

Stock solutions of Tz **1** and **2** were prepared at a concentration of 10 mM in DMSO. Dilution into MeCN, MeOH, PBS, and citrate-phosphate buffer (pH 4.0, 5.5, 6.0, 6.5, 7.0, 7.5, 8.0, and 8.5, 10 mM) gave solutions for UV–vis measurements at a tetrazine concentration of 50 μM (5 μL Tz stock solution + 995 μL solvent; DMSO content: 0.5%). UV–vis spectra were recorded on a Thermo Fisher Scientific NanoDrop One^C^ Microvolume UV–vis Spectrophotometer in cuvette mode at 25 °C.

#### Stopped-Flow Measurements

Stock solutions of ax-TCO-PEG_4_ (**3**) and *endo*-BCN-PEG_4_ (**5**) in DMSO were prepared at an approximate concentration of 100 mM. The exact concentration was determined by absorbance titration with Tz **2** (extinction coefficient 433 M^–1^cm^–1^ at 520 nm), quantifying the decrease in Tz absorbance upon reaction with **3** or **5**. The initial DMSO stock solution was diluted with MeCN, MeOH, citrate-phosphate buffer (pH 4.0, 5.5, 7.0, or 8.5, 10 mM), or PBS (pH 7.4) to prepare solutions for stopped-flow analysis at a final dienophile concentration of 1 mM (DMSO content: 1%). 10 mM stock solutions of **1**, **2**, and **4** were prepared in DMSO. Serial dilution into MeCN, MeOH, citrate-phosphate buffer (pH 4.0, 5.5, 7.0, or 8.5, 10 mM), or PBS (pH 7.4) gave solutions for stopped-flow analysis at a Tz concentration of 100 μM (DMSO content: 1%). In every stopped-flow experiment, the tetrazine and the dienophile reactant were dissolved in the identical solvent, so that combining the two reactant solutions in the stopped-flow cell did not change the solvent composition; the DMSO cosolvent content was kept at ≤ 1% in the final reaction mixture in all cases.

Stopped-flow measurements were performed using an SX20-LED stopped-flow spectrophotometer (Applied Photophysics) equipped with a 535 nm LED (optical path length 10 mm, full width at half-maximum 34 nm) to monitor the characteristic Tz absorption at 520–540 nm. The reactant syringes were loaded with Tz and dienophile solutions and the instrument was primed. Measurements were done in sextuplicate for each tetrazine. Reactions were conducted at 37 °C and recorded automatically at the time of acquisition.

Data sets were analyzed by fitting an exponential decay using Prism 10 (GraphPad) to calculate the observed pseudo-first-order rate constants, which were converted into second-order rate constants via division by the dineophile concentration (500 μM in the reaction).

### Computational Methods

All DFT calculations were performed using ORCA 5.0.4.^25^ The M06-2X functional^34^ was used in combination with the def2-TZVP^35^ and the def2/J auxiliary basis set. The RIJCOSX approximation was employed. TightSCF and defGrid3 were used for all calculations. The CPCM model was used to model solvent effects. Local minima were confirmed by the absence of imaginary frequencies. Transition states were characterized by exactly one imaginary frequency associated with the investigated reaction. All possible conformers were considered and lowest-energy values are reported here.

For TD-DFT, the first 30 excited states were calculated. To plot calculated UV–vis spectra, the orca_mapspc utility was used with a line width of 2000 cm^–1^.

Distortion/interaction analysis was performed in the gas phase using geometries optimized with solvation. The distortion energy (Δ*E*_dist_) was calculated as the difference in electronic energy between each isolated reactant in its transition-state geometry and its fully relaxed equilibrium geometry. The interaction energy (Δ*E*_int_) was obtained as the difference between the electronic energy of the transition state and the sum of the electronic energies of the distorted reactants at the transition-state geometry.

## Supplementary Material





## Notes

Computational data, additional UV–vis spectra, stopped-flow data, NMR data, and coordinates of calculated structures (ZIP)
(PDF)
