# Supplementary material for: Solvatochromism and Click Reactivity of Yellow Tetrazines
Source: Bioconjug Chem. 2026 Aug 27;37(9):1810–6. doi: 10.1021/acs.bioconjchem.6c00097 (PMC13587597; doi:10.1021/acs.bioconjchem.6c00097)
Supplement: Supplementary file 1 [file bc6c00097_si_002.pdf]

Supporting Information

Solvatochromism and Click Reactivity of Yellow Tetrazines

Patrick Keppel,[a] Jakob Raffler,[a] Hannes Mikula,[a] and Dennis Svatunek*[a]

[a] P. Keppel, J. Raffler, Dr. Hannes Mikula, Dr. Dennis Svatunek
Institute of Applied Synthetic Chemistry
TU Wien
Getreidemarkt 9, 1060 Vienna, Austria
E-mail: dennis.svatunek@tuwien.ac.at

Table of Contents

1 Computational Results 2

2 UV/vis spectra 4

3 Stopped-flow Measurements 7

4 NMR 12

5 References 15

1 Computational Results

Table S1 lists calculated energies of structures used in this study. Cartesian coordinates of all structures are provided as separate files.

Table S1. M06-2X/def2-TZVP CPCM(water) calculated energies in hartree.

structure	ΔE	ΔH	ΔG
**1**	-940.98959309	-940.77387047	-940.8310222
**2**	-790.51070458	-790.30642526	-790.3581926
**[1–H]$^-$**	-940.51658505	-940.31426713	-940.3706974
**TCO**	-313.20597743	-312.99264274	-313.0322295
**TS$_{1+TCO}$**	-1254.19636967	-1253.76519200	-1253.8360912
**TS$_{2+TCO}$**	-1103.71704422	-1103.29669566	-1103.3645426
**TS$_{[1-H]^-+TCO}$**	-1253.72074081	-1253.30321635	-1253.3738892

TD-DFT results for 1

```
-----------------------------------------------------------------------------
         ABSORPTION SPECTRUM VIA TRANSITION ELECTRIC DIPOLE MOMENTS
-----------------------------------------------------------------------------
State   Energy    Wavelength  fosc         T2        TX        TY        TZ
        (cm-1)      (nm)                 (au**2)    (au)      (au)      (au)
-----------------------------------------------------------------------------
   1   19035.4    525.3   0.006993755   0.12096   0.00014   0.34779  -0.00022
   2   28253.0    353.9   0.000200960   0.00234   0.00027   0.04839   0.00003
   3   29500.2    339.0   0.049087843   0.54780  -0.59697  -0.00008  -0.43752
   4   31501.6    317.4   1.155693541  12.07773   3.44184  -0.00084   0.48112
   5   33367.2    299.7   0.000000001   0.00000  -0.00008  -0.00007  -0.00000
   6   35440.1    282.2   0.000000010   0.00000  -0.00024   0.00002  -0.00018
   7   36822.0    271.6   0.000000001   0.00000   0.00003   0.00008  -0.00000
   8   37143.6    269.2   0.007791010   0.06905  -0.00004   0.26278  -0.00023
   9   38200.3    261.8   0.000000027   0.00000   0.00048  -0.00002   0.00003
  10   40862.8    244.7   0.000160650   0.00129  -0.00003  -0.03598   0.00002
  11   42489.9    235.3   0.008618794   0.06678  -0.20941   0.00002  -0.15141
  12   42867.6    233.3   0.000000031   0.00000   0.00038   0.00000   0.00030
  13   43724.3    228.7   0.210864591   1.58766   1.23113  -0.00066  -0.26826
  14   45138.2    221.5   0.000000001   0.00000  -0.00001  -0.00007   0.00000
  15   45743.8    218.6   0.000000004   0.00000  -0.00002  -0.00016  -0.00001
  16   46880.5    213.3   0.000000084   0.00000   0.00060   0.00002  -0.00048
  17   47071.6    212.4   0.001711124   0.01197   0.00026   0.10939  -0.00027
  18   48580.3    205.8   0.000000023   0.00000  -0.00007  -0.00038   0.00006
  19   48611.3    205.7   0.000194418   0.00132  -0.00039  -0.03628   0.00041
  20   48820.8    204.8   0.434706025   2.93134   1.34383  -0.00109  -1.06087
  21   49211.1    203.2   0.000000153   0.00000   0.00086  -0.00001  -0.00054
  22   50764.5    197.0   0.000000014   0.00000   0.00009  -0.00000  -0.00029
  23   52535.8    190.3   0.000000118   0.00000  -0.00001  -0.00086  -0.00001
  24   52727.6    189.7   0.000000015   0.00000   0.00024   0.00000  -0.00018
  25   53406.6    187.2   0.362097234   2.23206  -1.26257   0.00094   0.79873
  26   53518.2    186.9   0.002413200   0.01484   0.00030  -0.12184  -0.00015
  27   54773.7    182.6   0.230396459   1.38478   0.52301  -0.00087  -1.05415
  28   55262.6    181.0   0.001394362   0.00831   0.00027   0.09114  -0.00034
  29   55664.7    179.6   0.000000229   0.00000   0.00095  -0.00004  -0.00067
  30   56608.3    176.7   0.267555022   1.55600  -1.06921  -0.00009  -0.64248
```

TD-DFT results for [1–H]⁻

ABSORPTION SPECTRUM VIA TRANSITION ELECTRIC DIPOLE MOMENTS

State	Energy (cm-1)	Wavelength (nm)	fosc	T2 (au**2)	TX (au)	TY (au)	TZ (au)
1	18376.6	544.2	0.006450295	0.11556	0.04850	-0.30918	0.13270
2	22036.2	453.8	0.004804190	0.07177	0.12839	0.17015	0.16229
3	24922.3	401.2	0.567582386	7.49751	2.71488	0.17160	-0.31224
4	28080.9	356.1	0.097720065	1.14564	-1.06321	-0.09155	0.08276
5	31811.5	314.4	0.028610475	0.29608	0.53845	0.01619	-0.07678
6	32343.8	309.2	0.010015775	0.10195	-0.29208	0.12055	0.04585
7	34484.8	290.0	0.009776691	0.09333	0.12621	0.06144	0.27135
8	36399.9	274.7	0.348619966	3.15303	1.72255	-0.02797	0.43018
9	37690.7	265.3	0.004151487	0.03626	0.08475	-0.14120	0.09561
10	38322.4	260.9	0.042900535	0.36854	0.60258	0.04740	0.05652
11	39373.6	254.0	0.009845013	0.08232	0.28164	0.05355	0.01141
12	39640.8	252.3	0.006885259	0.05718	-0.19711	0.13538	-0.00070
13	42457.4	235.5	0.009550437	0.07405	-0.07676	-0.10898	-0.23724
14	43107.4	232.0	0.031512127	0.24066	0.43349	0.14212	-0.18042
15	43714.0	228.8	0.034753896	0.26173	-0.50397	0.00787	-0.08766
16	45095.8	221.8	0.009752522	0.07120	0.23126	-0.01913	0.13171
17	45582.2	219.4	0.032037709	0.23139	0.41754	-0.19494	-0.13803
18	45979.9	217.5	0.277079331	1.98386	-1.34853	0.19186	-0.35849
19	46203.4	216.4	0.070308280	0.50097	0.10847	-0.20286	0.66936
20	46687.3	214.2	0.070235787	0.49526	-0.50806	0.17452	-0.45463
21	47034.6	212.6	0.026632966	0.18641	-0.36380	0.12805	0.19408
22	47528.7	210.4	0.005654274	0.03916	-0.12617	0.09126	-0.12214
23	48549.3	206.0	0.031590405	0.21421	0.39636	-0.11901	0.20725
24	48746.7	205.1	0.156533987	1.05716	-0.63722	0.32206	-0.73985
25	50378.8	198.5	0.007604808	0.04970	-0.12565	0.13213	-0.12825
26	50510.5	198.0	0.064863770	0.42276	-0.53222	0.30521	-0.21529
27	51078.7	195.8	0.120328281	0.77554	0.30894	-0.03009	-0.82413
28	51413.9	194.5	0.110767680	0.70926	0.38848	-0.11621	-0.73813
29	52372.4	190.9	0.005913444	0.03717	-0.19025	-0.00621	0.03066
30	53184.3	188.0	0.004807652	0.02976	-0.15981	-0.03346	-0.05570

2 UV/vis spectra

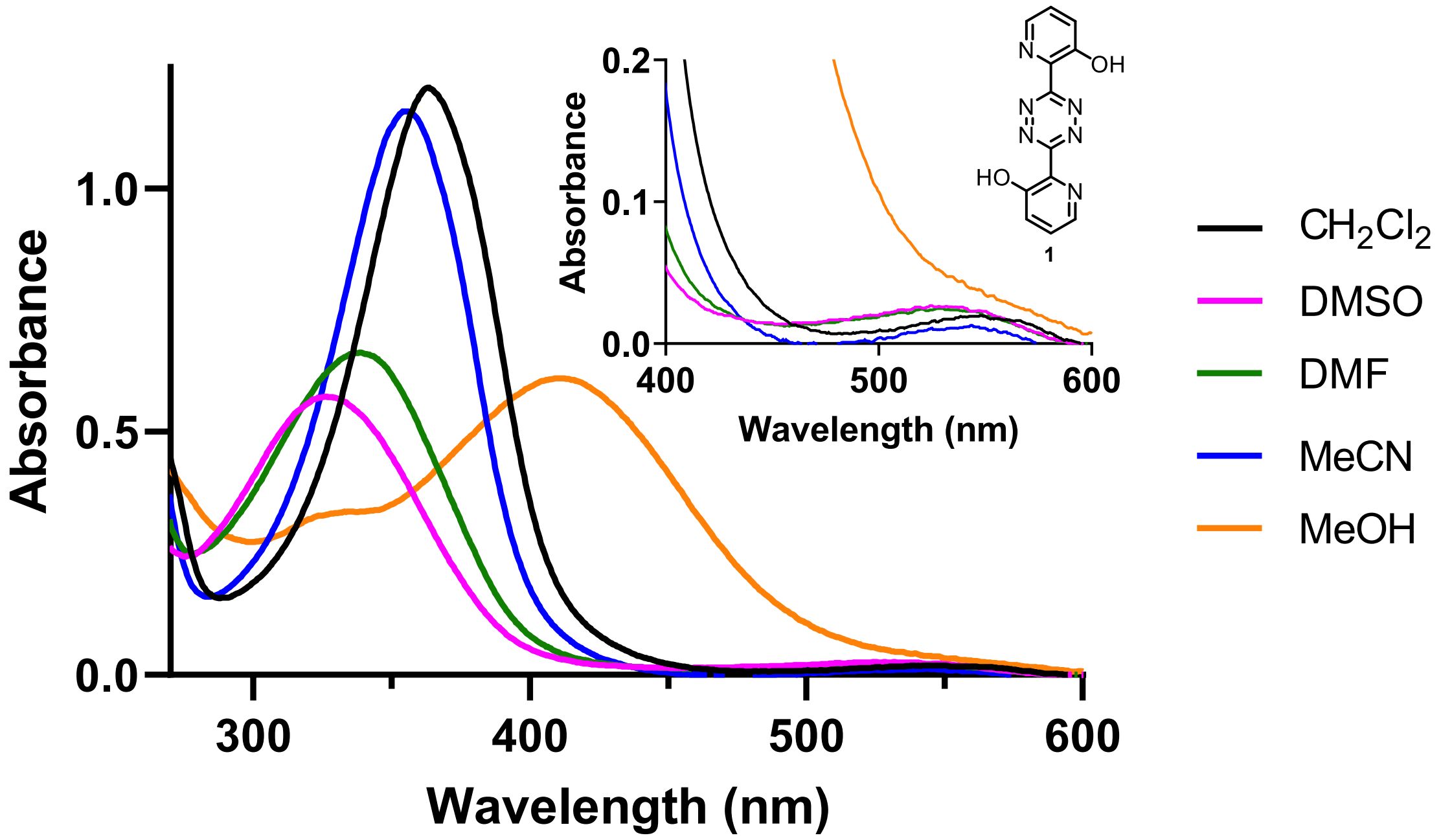

Figure S1. Absorption spectra of **1** in CH_2Cl_2, DMSO, DMF, MeCN, and MeOH showing solvatochromism.

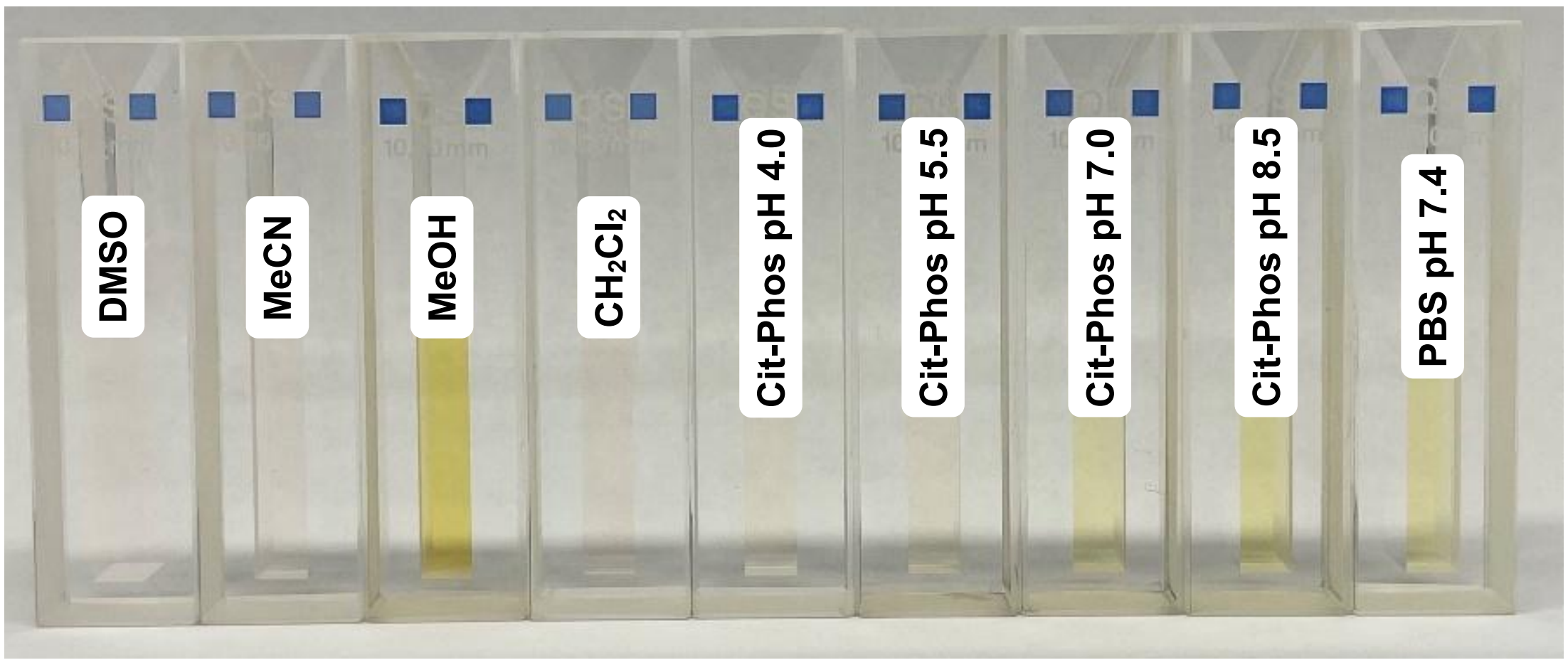

Figure S2. Solvatochromism of **1** in DMSO, MeCN, MeOH, CH_2Cl_2, citrate-phosphate buffer (Cit-Phos, pH 4.0, 5.5, 7.0, and 8.5, 10 mM), and PBS (pH 7.4).

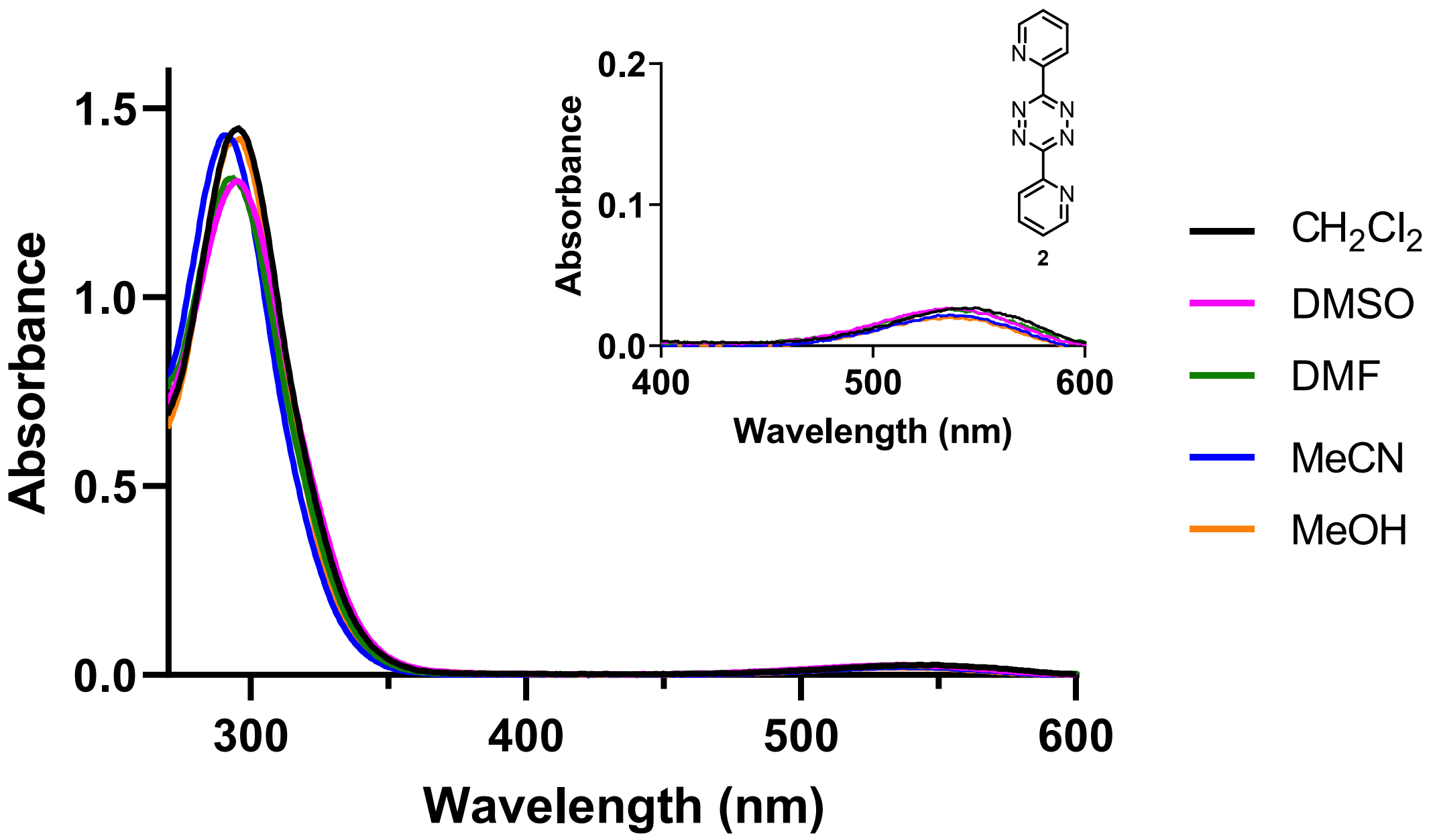

Figure S3. Absorption spectra of **2** in CH_2Cl_2, DMSO, DMF, MeCN, and MeOH showing no solvatochromism.

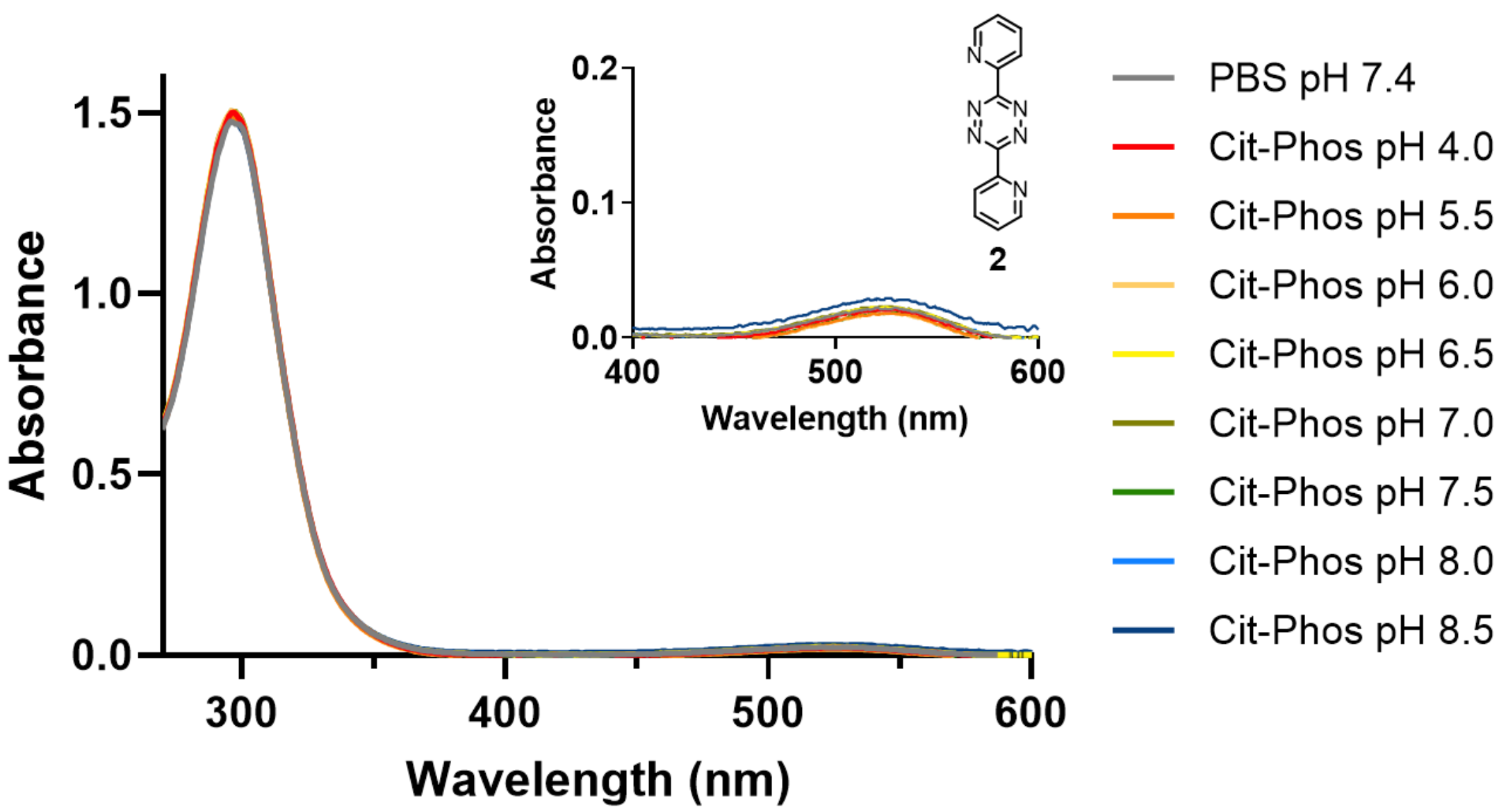

Figure S4. Absorption spectra of **2** in citrate-phosphate buffer (Cit-Phos, pH 4.0, 5.5, 6.0, 6.5, 7.0, 7.5, 8.0, and 8.5, 10 mM), and PBS (pH 7.4).

For pH-dependent reversible color change, bis-hydroxypyridyl Tz (**1**) was diluted into Cit-Phos buffer (pH 8.5) at a concentration of 50 µM (5 µL 10 mM Tz stock, 995 µL buffer, DMSO content: 0.5%). The UV/Vis absorption spectrum was recorded, followed by addition of 10 µL 1 M HCl and thorough mixing. A second UV/Vis absorption spectrum was recorded, and the pH of the solution was determined to be 4.0.

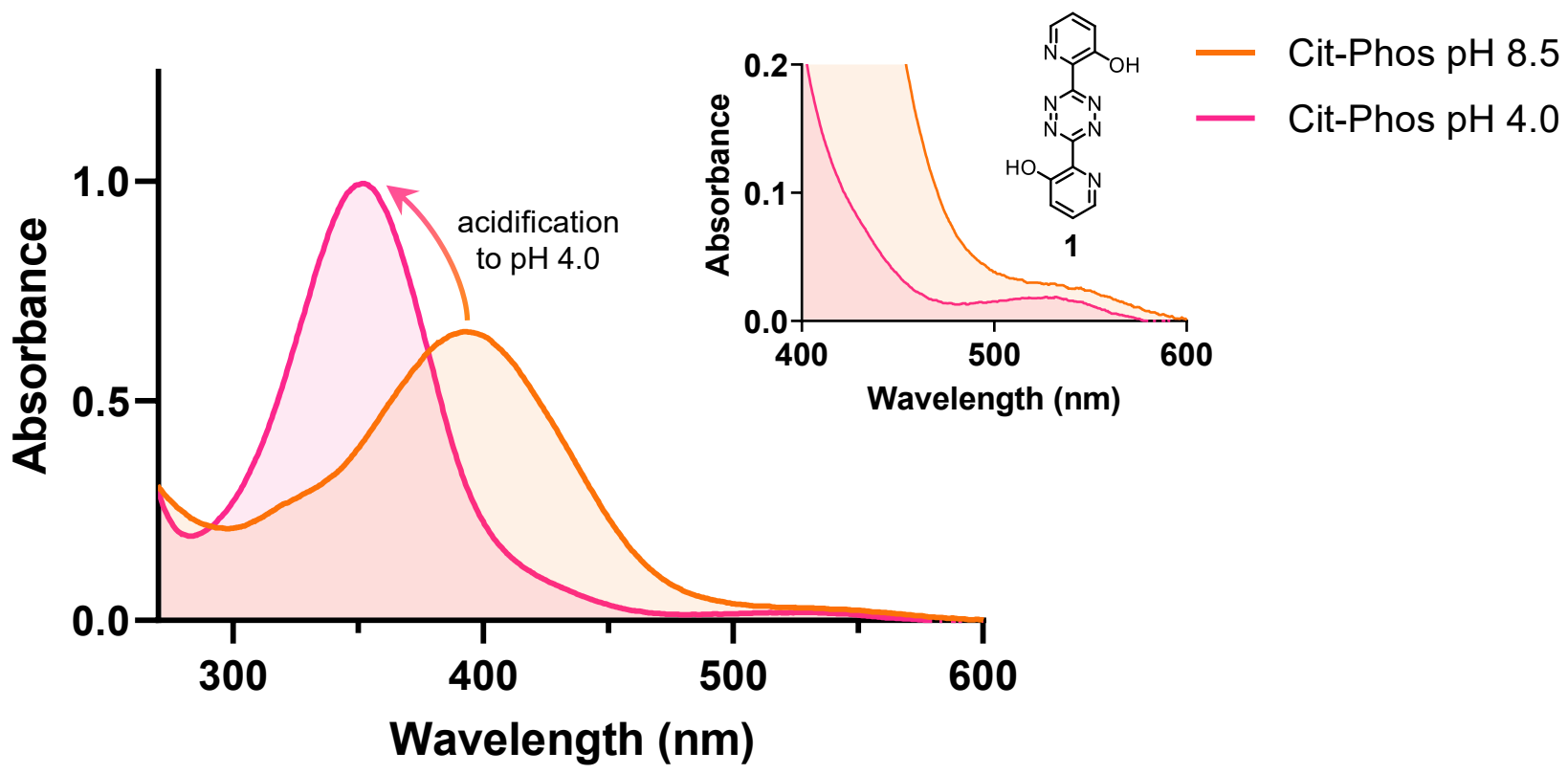

Figure S5. UV/Vis absorption spectra of **1** (50 µM) in Cit-Phos buffer (pH 8.5) and after addition of 10 µL 1 M HCl to adjust the pH to 4.0.

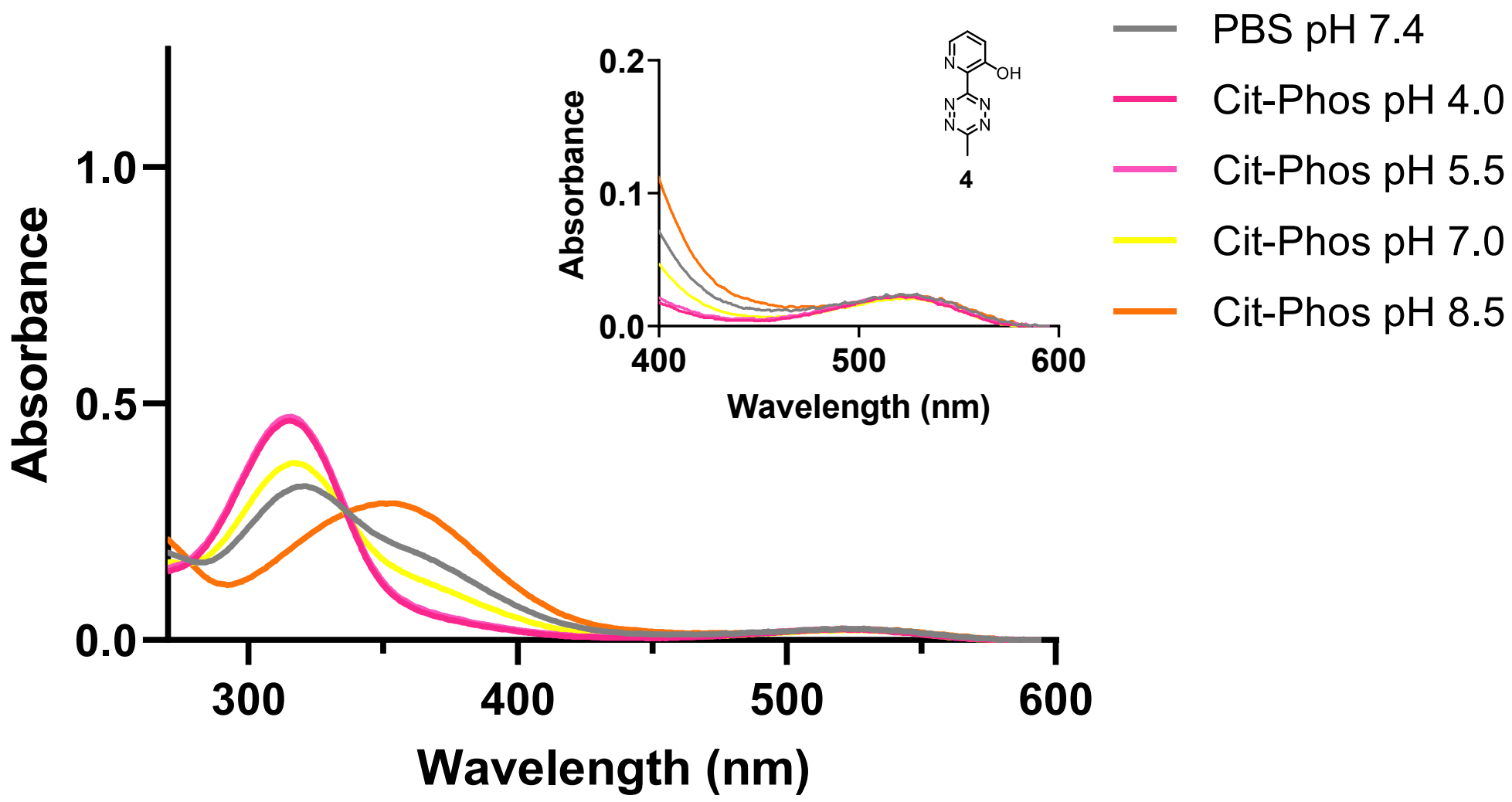

Figure S6. Absorption spectra of **4** in citrate-phosphate buffer (Cit-Phos, pH 4.0, 5.5, 7.0, and 8.5, 10 mM), and PBS (pH 7.4).

Table S2. Molar absorption coefficients (ε) of **1** in aqueous and organic solvents.

solvent	pH	ε ($M^{-1}cm^{-1}$)		
		400 nm	450 nm	530 nm
PBS	7.4	10,480	4,267	587
Cit-Phos buffer	4.0	4,393	753	420
Cit-Phos buffer	5.5	4,593	887	380
Cit-Phos buffer	6.0	5,380	1,440	587
Cit-Phos buffer	6.5	6,460	2,067	467
Cit-Phos buffer	7.0	8,607	3,333	567
Cit-Phos buffer	7.5	10,880	4,500	640
Cit-Phos buffer	8.0	11,993	4,720	533
Cit-Phos buffer	8.5	12,813	4,840	640
CH_2Cl_2		7,120	453	340
DMSO		1,080	293	513
DMF		1,620	280	493
MeCN		3,567	100	200
MeOH		11,833	8,307	1,007

The isosbestic point was determined at 380 nm with an ε of 11,473 L mol^{-1} cm^{-1}.

3 Stopped-flow Measurements

Table S3. Second-order rate constants (k_2) ± 6%* of tetrazines **1**, **2**, and **4** with axTCO-PEG_4 (**3**) and *endo*-BCN-PEG_4 (**5**). All measurements were performed at 37 °C.

dienophile	solvent/buffer	pH	k_2 of **1** ($M^{-1}s^{-1}$)	k_2 of **2** ($M^{-1}s^{-1}$)	k_2 of **4** ($M^{-1}s^{-1}$)
axTCO-PEG_4 (**3**)	MeCN	-	910	790	n.d.
"	MeOH	-	150	1,700	n.d.
"	Cit-Phos	4.0	38,000	74,000	5,100
"	Cit-Phos	5.5	34,000	76,000	5,000
"	Cit-Phos	7.0	20,000	75,000	3,600
"	PBS	7.4	12,000	77,000	2,700
"	Cit-Phos	8.5	2,200	77,000	840
endo-BCN-PEG_4 (**5**)	Cit-Phos	4.0	1,100	1,500	n.d.
"	Cit-Phos	5.5	1,000	1,400	n.d.
"	Cit-Phos	7.0	560	1,500	n.d.
"	PBS	7.4	380	1,500	n.d.
"	Cit-Phos	8.5	110	1,400	n.d.

*** Uncertainties of TCO titration and stopped-flow analysis were evaluated experimentally from the dispersion of repeated measurements and expressed as standard deviation. The reported uncertainty is an expanded uncertainty calculated using a coverage factor of 2 which gives a level of confidence of approximately 95%.[1]**

Kinetic data with exponential fits

37 °C, DMSO content: 1%

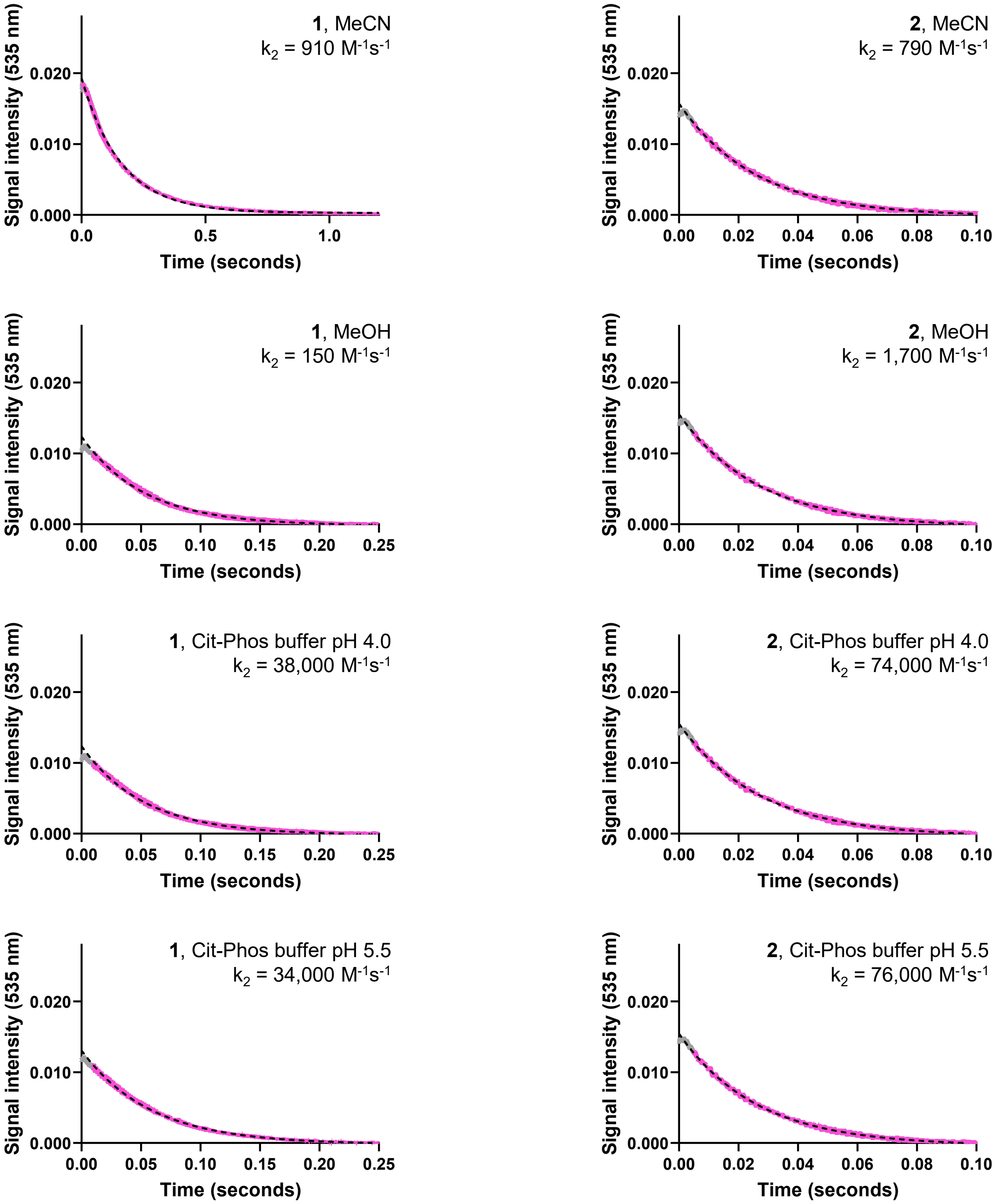

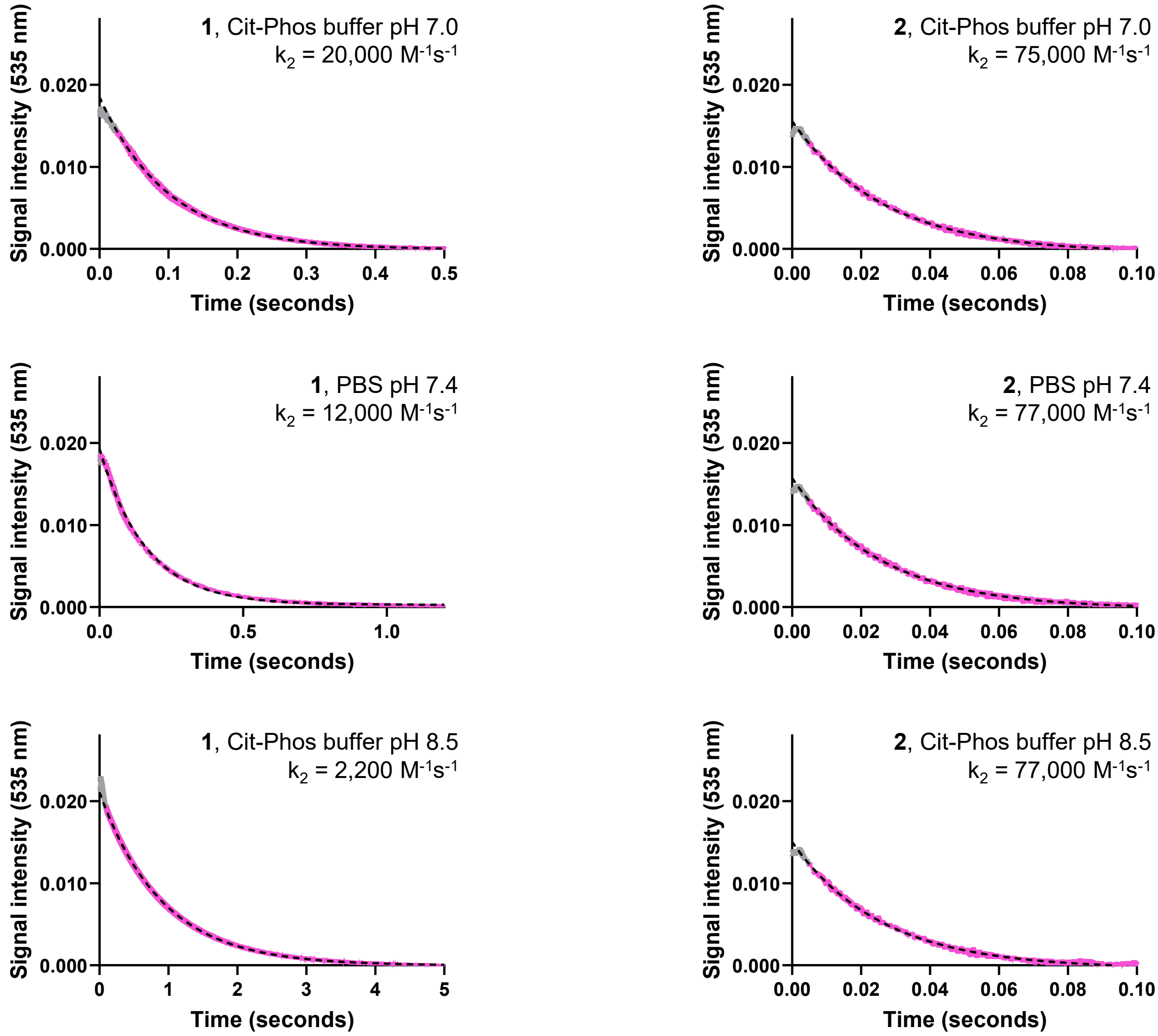

Figure S7. Stopped-flow absorbance traces for reactions of axTCO-PEG_4 (**3**) with Tz **1** and **2** in MeCN, MeOH, citrate-phosphate buffer and PBS, recorded under pseudo-first-order conditions (500 µM TCO, 50 µM Tz, *n* = 6). The curves represent the measured stopped-flow traces, the pink-highlighted data were used for the determination of k_2 and the dashed lines indicate the non-linear regression analysis.

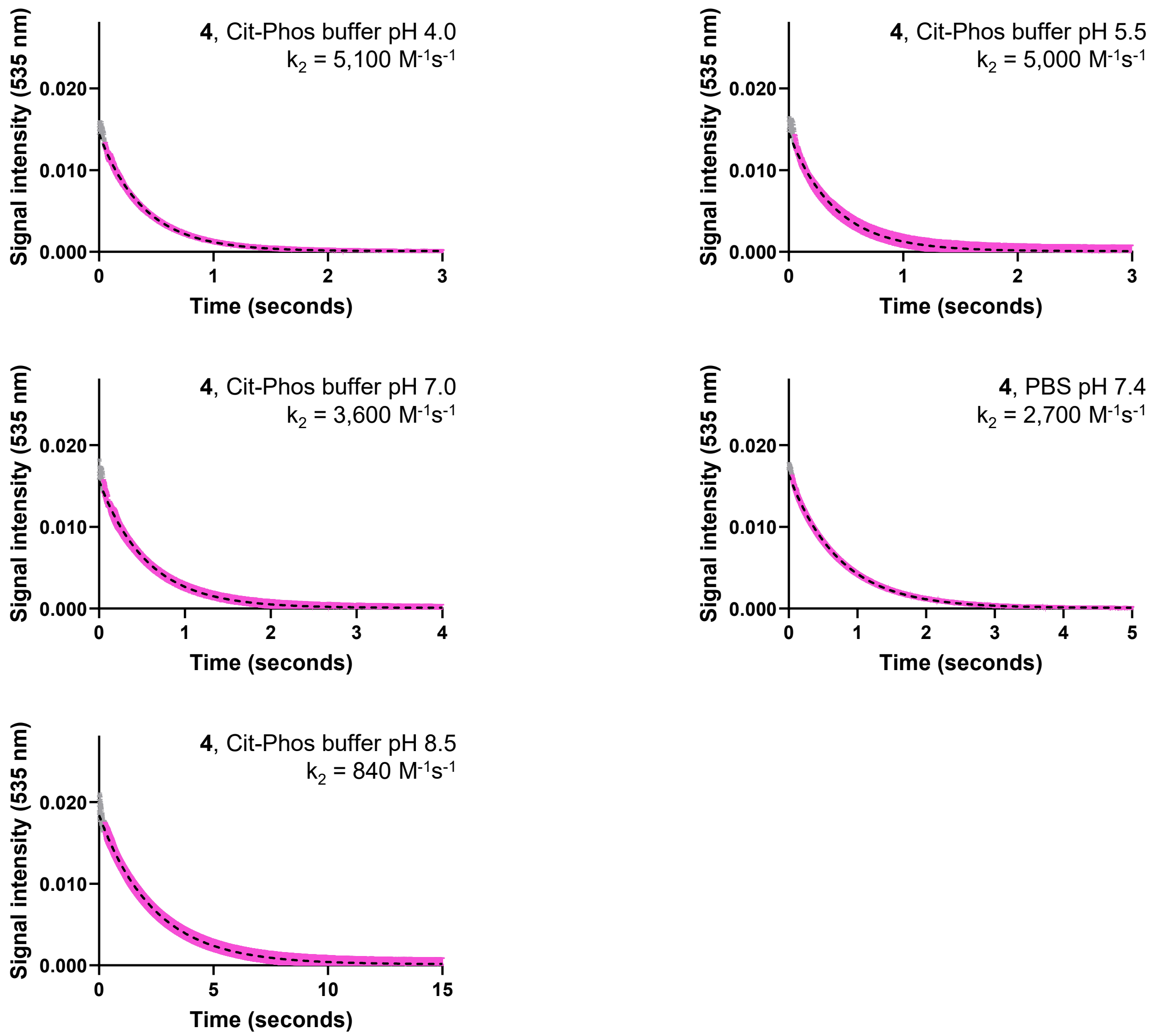

Figure S8. Stopped-flow absorbance traces for reactions of axTCO-PEG$_4$ (**3**) with Tz **4** in citrate-phosphate buffer and PBS, recorded under pseudo-first-order conditions (500 µM TCO, 50 µM Tz, *n* = 6). The curves represent the measured stopped-flow traces, the pink-highlighted data were used for the determination of k_2 and the dashed lines indicate the non-linear regression analysis.

The reaction rate constants of **1** with **3** were also determined in degassed, oxygen-free PBS (sonicated while purged with argon for 20 minutes) and in oxygenated PBS (O_2 bubbled through the solution for 20 minutes). The resulting traces are shown in Figure S9. Identical reactivity was observed in both cases, with the rate constant determined to be 11,000 $M^{-1}s^{-1}$.

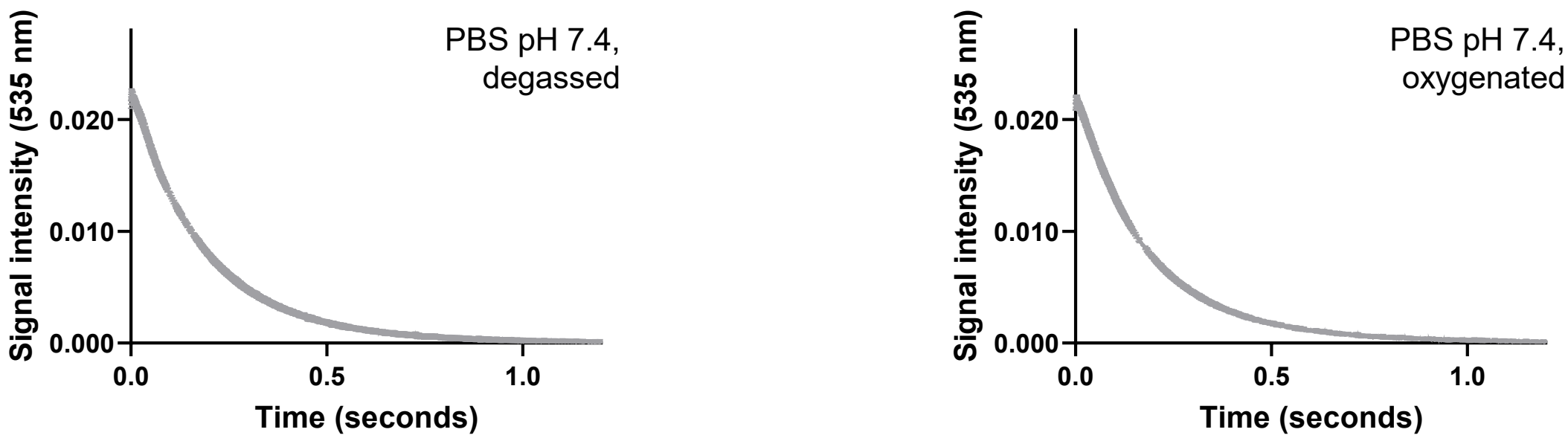

Figure S9. Stopped-flow absorbance traces for reactions of axTCO-PEG$_4$ (**3**) with Tz **1** in degassed and oxygenated PBS, recorded under pseudo-first-order conditions (500 µM TCO, 50 µM Tz, n = 6). No relevant difference was observed between the traces recorded in degassed and oxygenated PBS.

4 NMR

axTCO-PEG$_4$ (**3**), ^{1}H NMR (400 MHz)

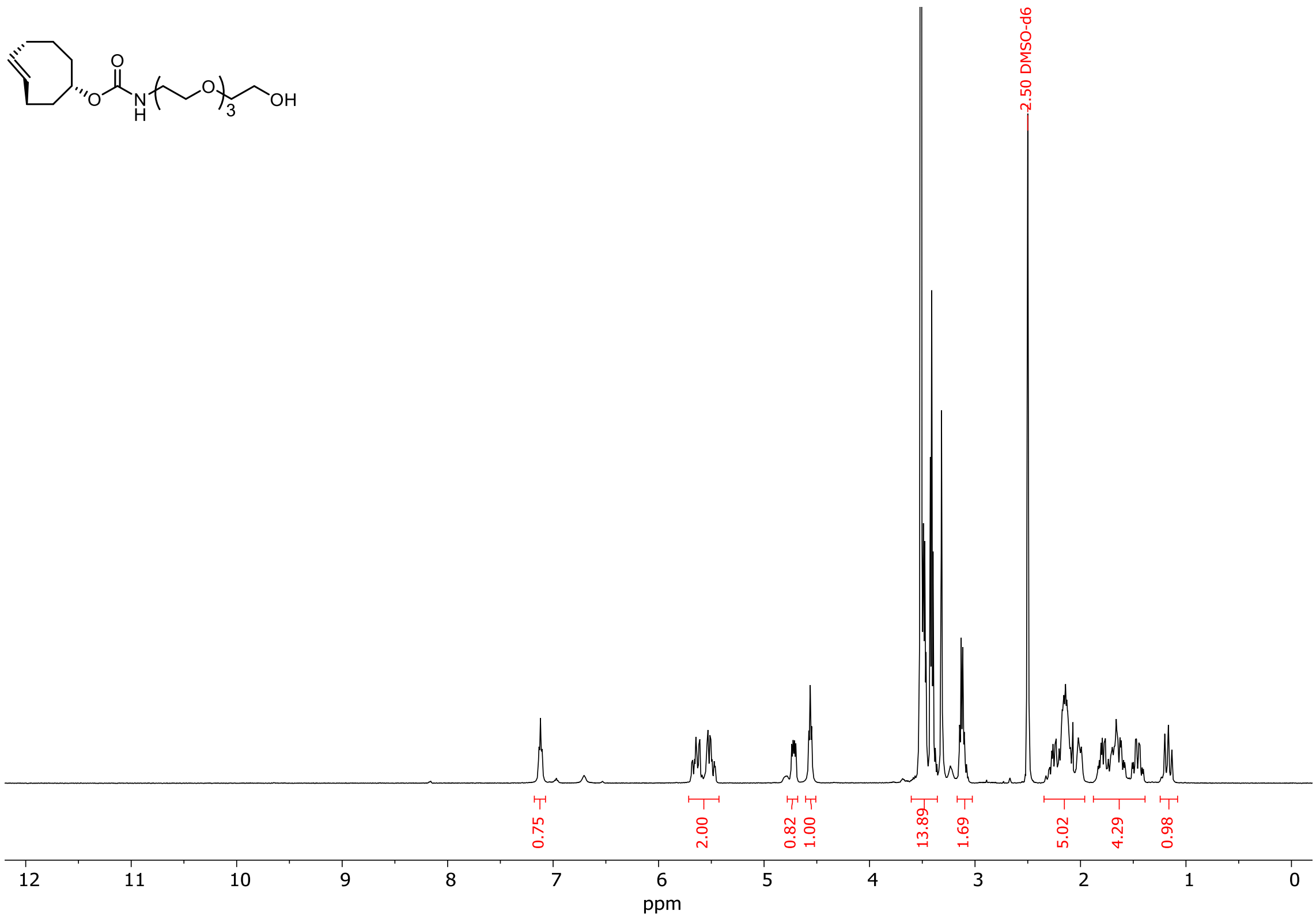

axTCO-PEG$_4$ (**3**), ^{13}C NMR (100 MHz)

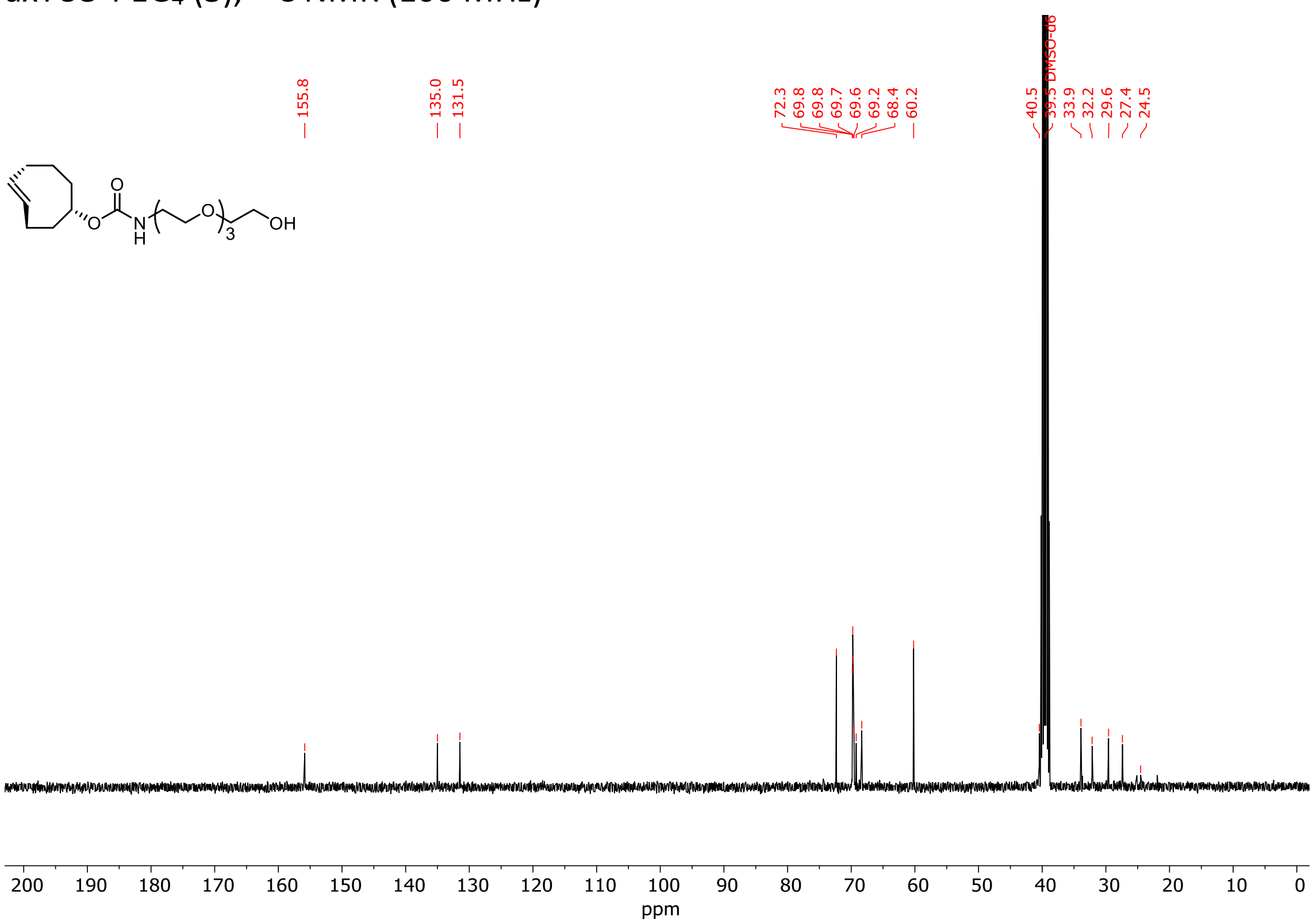

Methyl-hydroxypyridyl-Tz (**4**), ^{1}H NMR (600 MHz)

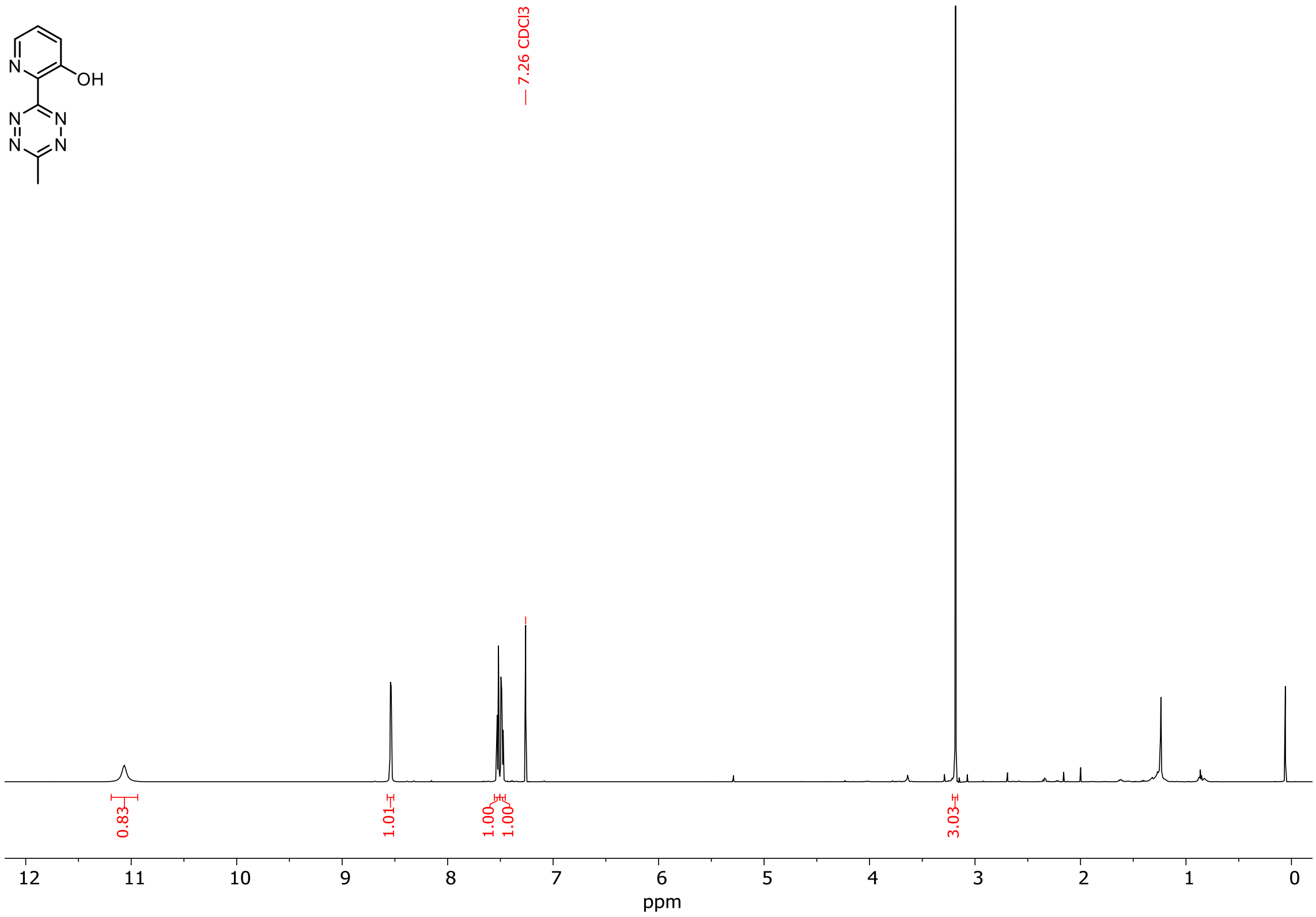

Methyl-hydroxypyridyl-Tz (**4**), ^{13}C NMR (151 MHz)

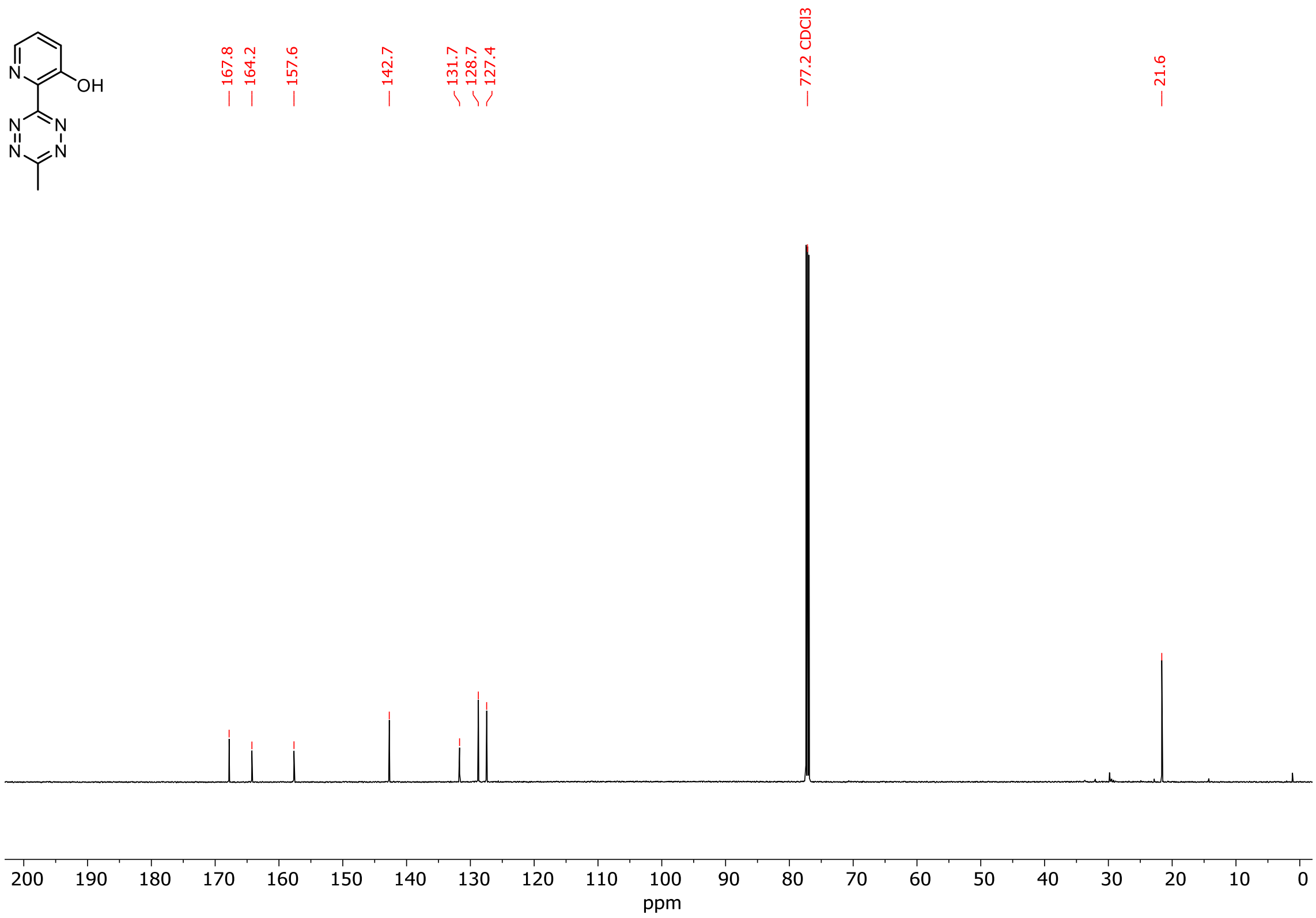

endo-BCN-PEG$_4$ (**5**), ^{1}H NMR (600 MHz)

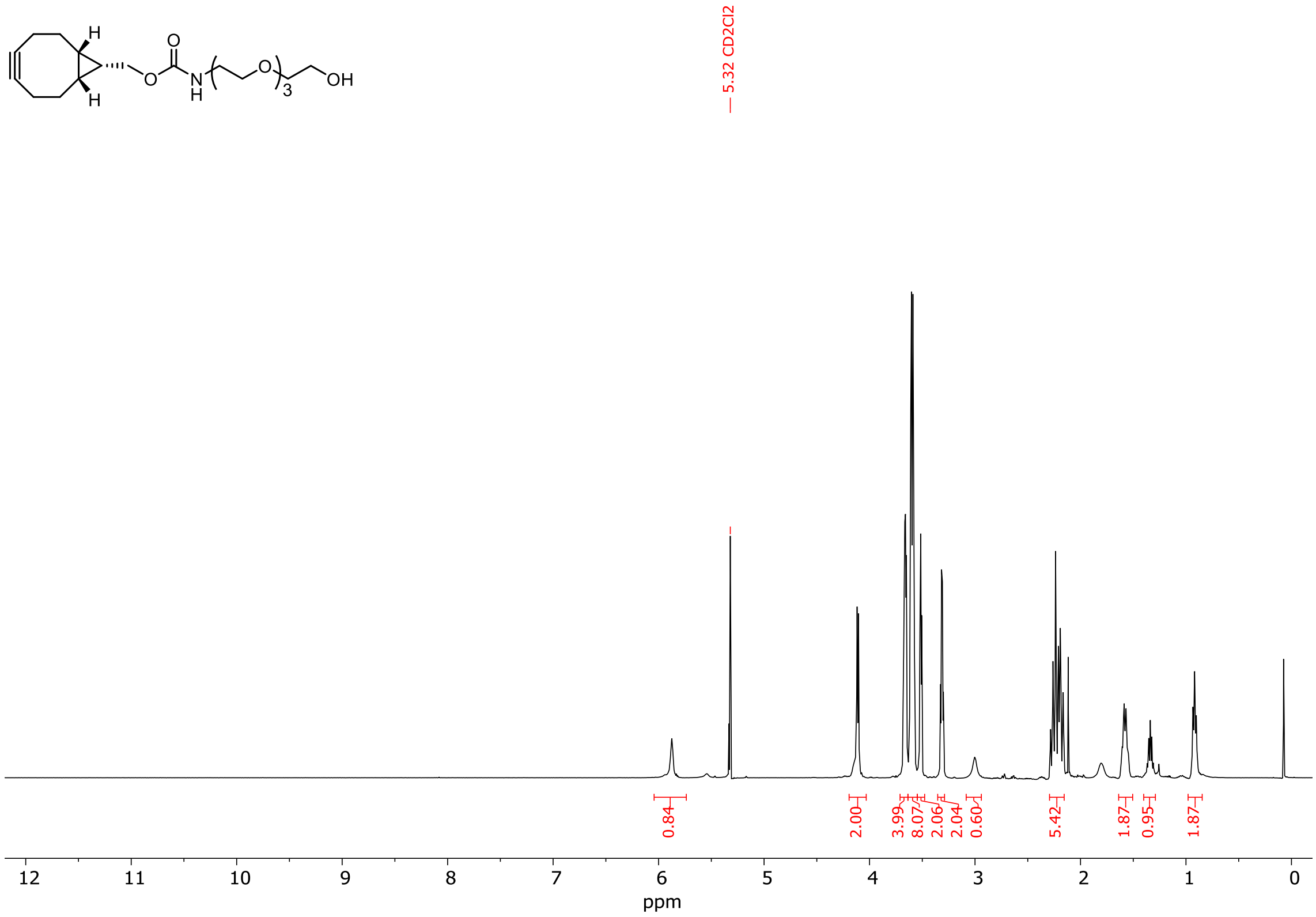

endo-BCN-PEG$_4$ (**5**), ^{13}C NMR (151 MHz)

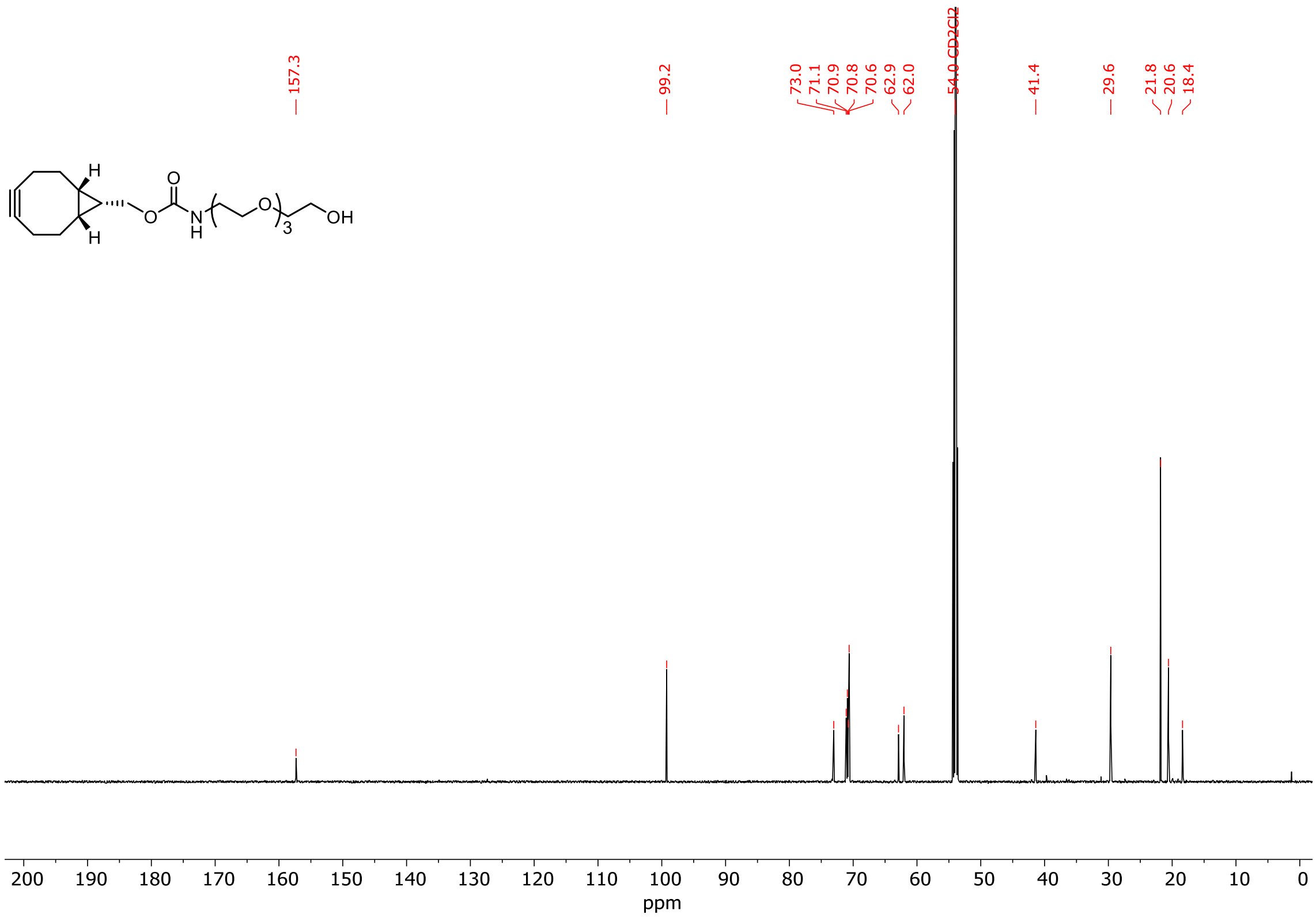

5 References

[1] S. L. R. Ellison, A. Williams, *Quantifying Uncertainty in Analytical Measurement*, 3rd ed., **2012**.
